# The Meso-Expression Test (MET): A Novel Assessment of Emotion Perception

**DOI:** 10.3390/jintelligence11070145

**Published:** 2023-07-19

**Authors:** Matthew L. LaPalme, Sigal G. Barsade, Marc A. Brackett, James L. Floman

**Affiliations:** 1 Yale Center for Emotional Intelligence, Yale University, New Haven, CT 06511, USA; marc.brackett@yale.edu (M.A.B.);; 2 Wharton, University of Pennsylvania, Philadelphia, PA 19104, USA

**Keywords:** emotion, emotional intelligence, assessment, EI

## Abstract

Emotion perception is a primary facet of Emotional Intelligence (EI) and the underpinning of interpersonal communication. In this study, we examined meso-expressions—the everyday, moderate-intensity emotions communicated through the face, voice, and body. We theoretically distinguished meso-expressions from other well-known emotion research paradigms (i.e., macro-expression and micro-expressions). In Study 1, we demonstrated that people can reliably discriminate between meso-expressions, and we created a corpus of 914 unique video displays of meso-expressions across a race- and gender-diverse set of expressors. In Study 2, we developed a novel video-based assessment of emotion perception ability: The Meso-Expression Test (MET). In this study, we found that the MET is psychometrically valid and demonstrated measurement equivalence across Asian, Black, Hispanic, and White perceiver groups and across men and women. In Study 3, we examined the construct validity of the MET and showed that it converged with other well-known measures of emotion perception and diverged from cognitive ability. Finally, in Study 4, we showed that the MET is positively related to important psychosocial outcomes, including social well-being, social connectedness, and empathic concern and is negatively related to alexithymia, stress, depression, anxiety, and adverse social interactions. We conclude with a discussion focused on the implications of our findings for EI ability research and the practical applications of the MET.

## 1. Introduction

### 1.1. Emotion Perception as the Primary Facet of Emotional Intelligence

Emotional Intelligence (EI) is defined as the ability to perceive, understand, use, and regulate emotions in both the self and others (Salovey and Mayer 1990). In their seminal work, Salovey and Mayer (1990), coined the term EI and helped spark an affective revolution (Barsade et al. 2003) that centered emotions and emotion-related abilities as a central domain in psychology. In the three decades since then, EI research and the assessment of EI rapidly increased.

While EI consists of multiple facets, prior research has demonstrated that emotion perception demonstrates primacy and is key to the function of other emotion abilities (Joseph and Newman 2010). That is, accurately perceiving emotion facilitates emotion understanding, use, or regulation (Joseph and Newman 2010; Gregory et al. 2020). The ability to perceive emotions is so important that it occurs across cultures (Cordaro et al. 2018; Ekman et al. 1987, 1969; cf. Matsumoto 1992), and is the underpinning of interpersonal communication (Fischer and Manstead 2016; Mehrabian 1971; Mehrabian and Ferris 1967) because it conveys fitness enhancing messages (Tracy and Robins 2008a). Some scholars have even gone as far to say that most information is communicated non-verbally through facial expressions and body language (Mehrabian 1971, 2017). That said, the ability to accurately perceive emotions predicts a broad array of life, work, and social outcomes (Mayer et al. 2008). Emotion perception ability is positively associated with healthy supportive social relationships and relationship quality (Brackett et al. 2006; Brazeau 2021; Hall et al. 2009). Emotion perception is also associated with well-being and life satisfaction (Brackett et al. 2006), academic and workplace performance (Hall et al. 2009; MacCann et al. 2020), and psychological adaptation (e.g., cultural adjustment when moving to a new country; Hall et al. 2009). Given the importance for communicating effectively and building relationships, emotion perception is the EI ability we focus on in the current study. 

In this research, we sought to develop a novel assessment of emotion perception: the Meso-Expression Test (MET). This study aims to improve upon prior emotion perception assessments by focusing on meso-expressions, which we theorize are the moderate-intensity, consciously expressed, ecologically valid emotion expressions of everyday life. We ground this meso-expression paradigm in the componential theory of emotions, which views them in terms of their shared characteristics and components (e.g., appraisals, feelings, expressions, physiological patterns, and action tendencies/behaviors; Dael et al. 2012; Shaver et al. 1987). 

We address prior measurement shortcomings as well by using racially diverse expressors in our stimuli, and racially diverse perceivers in our validation studies—and applying robust analyses of measurement bias (i.e., measurement equivalence; Vandenberg and Lance 2000) in the MET. Finally, our study also addresses several methodological critiques of ability EI assessments, including the methods used to elicit emotion displays, as well as the methods used to label emotions and judge correctness. The MET advances EI research by helping researchers better measure how people perceive emotions in daily life, with more ecological validity and less intergroup bias. Below, we define what meso-expressions are and why they are important, and we elaborate on the theoretical and practical advancements this paper aims to fulfill in developing the MET.

### 1.2. Meso-Expressions

We coin the term *meso-expression* to describe moderate-intensity, conscious, everyday expressions of emotion. Prior literature and assessments of emotion^1^ have focused primarily on prototypical *macro-expressions* (Matsumoto and Hwang 2014), or virtually undetectable *micro-expressions* (Ekman 2009; Matsumoto and Hwang 2011) and we begin by contextualizing meso-expressions within this body of prior literature.

Macro-expressions are prototypical displays of emotion that are high in emotional intensity, may last several seconds, are often consciously displayed in order to convey feelings, and are easy to identify (Ekman 2003). A substantial portion of prior emotion perception research and assessments has focused on such expressions, likely because they are more readily recognized across diverse sets of perceivers, and the high recognizability of emotional macro-expressions has been used as evidence of emotion universality (Ekman 2003; Ekman et al. 1987, 1969). Several scholars have critiqued the validity of using macro-expressions in research on emotion perception, as macro-expressions are confounded by artificiality and they suffer from a lack of ecological validity (Barrett 2017a; Buck et al. 2017). That is, while macro-expressions certainly are a part of the construct space of emotions, they are less common expressions; therefore, the focus on examining macro-expression does not adequately capture or represent the spectrum of emotions people must perceive in everyday life.

Micro-expressions by comparison are brief, low-intensity expressions that are difficult to reliably perceive and interpret (Ekman 2009; Matsumoto and Hwang 2011; Yan et al. 2013). Micro-expressions also occur involuntarily and may be activated by separate neural pathways in comparison to macro-expressions (Rinn 1984; Tamietto and De Gelder 2010). Research on micro-expressions has focused on naturally occurring and unconscious displays of emotion as a way to perceive unconscious emotion states and even detect lies (Ekman 2009). Putatively, this approach may confer greater ecological validity than posed macro-expressions. That said, because micro-expressions are displayed outside of conscious awareness, research has framed the perception of micro-expressions as a form of psychological eavesdropping (Elfenbein and Ambady 2002b), and demonstrated that, somewhat counterintuitively, the ability to perceive micro-expressions may be negatively related to adaptive social functioning (if at all) (Blanck et al. 1981; Elfenbein and Ambady 2002a; Puccinelli and Tickle-Degnen 2004). Relatedly, training to detect micro-expressions has not been found to improve the ability to detect lies in others (Jordan et al. 2019; Warren et al. 2009) despite the popular appeal of using micro-expressions for this purpose (Ekman 2009) and scholars have noted their limited utility (Matsumoto and Hwang 2011). As such, micro-expression emotion perception measurement paradigms confer limited predictive validity, specifically when it comes to the purported range of social and psychological benefits enjoyed by those skilled at emotion perception (e.g., Mayer et al. 2016).

In the present research, we focus on an unexamined form of emotion display we coin *meso-expressions* which we theoretically distinguish from macro-expressions and micro-expressions. Meso-expressions are less intense (see also subtle emotions: Matsumoto and Hwang 2011, 2014; Warren et al. 2009) and less transparent than macro-expressions (and are therefore more nuanced and common to everyday experience), but they are more intense and last longer than micro-expressions (and therefore may be more reliably detected and employed as predictive measures of emotion perception ability). Furthermore, we define meso-expression as typically occurring within the awareness of expressor (i.e., expression occurs consciously), which is important since, as noted above, eavesdropping on unconscious micro-expressions is not a reliable indicator of social-emotional functioning. Meso-expressions may not follow the regimented activation patterns associated with macro-expressions (i.e., they are not emotion prototypes), and therefore, they may be more nuanced and naturalistic (i.e., they better match the diversity of emotion we perceive in everyday life). Research shows that expression prototypicality and intensity are linearly related to recognition rates (Matsumoto and Hwang 2014). Thus, meso-expressions may be harder to detect and challenge emotion perception ability more than macro-expressions. Finally, given that meso-expressions may more closely represent the types of emotion displays we see in everyday life (Matsumoto and Hwang 2011, 2014), we argue that assessing the ability to perceive meso-expressions is a more ecologically valid test of emotion perception ability. 

### 1.3. Emotions Are a ‘Fuzzy Set’ 

In the present work, when we refer to ‘emotions,’ we are referring to interrelated and continuous concepts of emotion which are considered a ‘fuzzy set’ (Cowen and Keltner 2017). The idea of emotions as a fuzzy set has long been theorized in the literature and, more recently, empirically demonstrated. Early research consistently demonstrated that emotion concepts describe highly inter-related phenomena and that the experience and features of emotion words are highly correlated (Dael et al. 2012; Russell 1980; Shaver et al. 1987; Watson et al. 1988)—which means one cannot describe emotions as independent fingerprints (cf. Barrett 2017a, 2017b). Furthermore, some scholars have argued that no two emotion instances may be the same (Barrett 2017a). For example, even the most common visible cues of fear, such as sweating (25% of individuals), trembling (30% of individuals), or screaming (23% of individuals), occur in less than a third of individuals as a typical expression (Shaver et al. 1987). 

More recent work has empirically demonstrated that emotions are ‘fuzzy’. Cowen and Keltner (2017) demonstrated that emotions are better modeled as having smooth gradients of relatedness that are mapped into a semantic space. A semantic space maps emotion terms along “their distinct varieties of reported experience” (p. 7906). In this critical study, they demonstrate that “the boundaries between many distinct emotions categories are fuzzy rather than discrete” (p. 7093). In fact, their study finds that most emotion categories share fuzzy boundaries with one or two categories of emotion, and may form fuzzy ‘chains’ of related experience (i.e., emotion families). For example, they find smooth boundaries that relate calmness to aesthetic appreciation which in turn shares a boundary with awe (URL Accessed 1 July 2023 https://s3-us-west-1.amazonaws.com/emogifs/map.html). As another example, the emotion concept of love shares some similarities and overlap with the emotion concept of sympathy (Cowen and Keltner 2020), and one could describe sympathy as a ‘shade’ on the gradient of love or vice-versa. Large-scale empirical research by the same researchers has also utilized deep neural networks to categorize millions of emotional responses and has found reliable patterns of interlinked emotion concepts (Cowen and Keltner 2021). This research directly supports the view that emotions are a fuzzy set without sharp boundaries or distinctions across a wide range of cultures around the world, but categories of emotion nevertheless exist and can be distinguished. Finally, research in human cognition also shows that modeling using fuzzy approaches to emotion classification may be adaptive as they result in higher emotion perception accuracy rates. For example, studies training computer models to use fuzzy approaches can result in accuracy of classification of emotion above 90% (Liliana et al. 2019, p. 391). Some have theorized that fuzzy logic results in better perception accuracy because fuzzy systems can handle ‘partial truth’ where there are degrees of difference between emotion categories. That is, emotions are complex, and each unique expression holds a degree or probability of ‘truth’ to every emotion label, and fuzzy logic is able to capture this variability (Liliana et al. 2019, p. 393; Zadeh 1965).

Based on the consistent evidence of the inter-relatedness of emotion concepts and variation within and between emotion concepts, we take the view that emotions are a *fuzzy set* (Cowen and Keltner 2017; Shaver et al. 1987) which means that emotions are separated by “vague rather than sharp boundaries” (Shaver et al. 1987, p. 1062). The consequences of viewing emotions as a fuzzy set are fourfold. First, componential emotion theory (Dael et al. 2012) posits the existence of many potential emotional states, and so we seek not only to measure the perception of putative ‘basic emotions,’ but also to measure the perception of a variety of additional emotions. Specifically, we constructed our novel test of emotion perception to capture displays of: amusement, anger, anxiety, awe, boredom, contempt, content, disgust, embarrassment, fear, joy, pride, relief, sadness, shame, surprise, and sympathy. All of these emotions have expressions with demonstrated recognizable features across cultures (Cordaro 2014; Cordaro et al. 2020; Cowen et al. 2021; Ekman and Friesen 1971, 1986; Izard 1971; Keltner 1995; Perkins et al. 2012; Tracy and Robins 2008b). That said, we treat the emotions included in the MET not as basic emotion fingerprints, but instead as a complex set of interrelated emotion family members that exist on gradients (i.e., fuzzy sets of emotion concepts: Cowen and Keltner 2021; Shaver et al. 1987). Therefore, when we refer to emotion perception, we are referring to distinguishing between and among emotions with emotions defined as fuzzy sets.

The second consequence of viewing emotions as fuzzy sets is that it is important to capture the broad array of non-verbal behaviors that coincide with each emotion. As such, we employed a multimodal approach which uses audio and video recordings of emotion expressions to capture vocal, facial, and bodily components of each emotion. Prior work taking the basic emotion view has primarily focused on facial movements. Consistent with more recent approaches (Cowen and Keltner 2017, 2020, 2021; Cowen et al. 2021), we also examine expression of emotion through bodily movement and through vocal expression (Dael et al. 2012; Laukka and Elfenbein 2012; Shaver et al. 1987). We aimed to capture multiple channels through which individuals may convey emotions, which is more consistent with the multi-faceted richness that characterizes the phenomenon of emotion perception in the real world. This approach helps to address the critique that tests of emotion perception may not adequately capture the nuance, complexity, and correspondent authenticity of emotion expression because of the narrow focus on facial movements alone (Dael et al. 2012; Elfenbein and Ambady 2002a). 

Third, due to viewing emotions as a fuzzy set, there are many possible patterns of activation that may constitute and/or be used to reliably express an emotion. As such, in the creation of the MET stimuli, we elicited emotion displays using autobiographical recall, as opposed to enforcing a fixed pattern of action units (cf. Ekman et al. 1987). Autobiographical recall is a form of method acting in which an individual takes time to recall and imagine being back in a moment when they distinctively experienced a particular emotion state (Stanislavski 1989). Recalling one’s memory of an emotion event simulates the autonomic nervous system activity, and phenomenology associated with that emotion (i.e., memory of an emotion activates that emotion: Coan and Allen 2007; Levenson et al. 1991) can result in spontaneous displays of the recalled emotion (Ekman 2003; El Haj et al. 2016) and captures the natural variability of emotion expressions. This approach helps to address the artificiality of using posed displays from individuals who may not be experiencing the emotions they are expressing (Barrett 2017a; Buck et al. 2017), which some have argued do not actually measure emotion perception accuracy because the expresser does not actually feel the emotion (Buck et al. 2017). 

Finally, viewing emotions as a fuzzy set within the componential theory of emotion also means that ‘labels’ assigned to consciously identify emotions may overlap, as they may reflect shared phenomenology, appraisal features, action tendencies, and/or expressive features (Cowen and Keltner 2017, 2021; Ekman 1997; Laukka and Elfenbein 2012; Scherer and Ellgring 2007; see also Vigliocco et al. 2014), and so we examine the relationships among emotion labels in the MET. As an example, disgust and anger are both considered high-activation negative emotions (Russell 1980; Cowen and Keltner 2017) whose action tendencies generally include the furrowing of the brow and tightening of the eyelids (Cordaro 2014; Cordaro et al. 2020). As these two emotions also share phenomenological similarities, they may be confused with one another (i.e., disgust may share enough similarities with anger that perceivers may reasonably confuse a disgust display for an anger display). Notably, selecting ‘anger’ for a disgust display still demonstrates higher emotion perception ability than selecting ‘amusement’ (an emotion that shares few if any characteristics with anger). Labels considered ‘incorrect’ may provide valuable information about the emotion perception ability of perceivers when emotions are viewed as a fuzzy set. Prior research supports this perspective and has also shown that emotion expressions may convey more than one emotion (Ekman et al. 1987; Barrett et al. 2019; Cowen and Keltner 2020, 2021). Emotion displays may be blended, for example, or may simply have multiple concurrent emotions where one expression is more salient than the others. For example, displays of disgust are often blended with contempt, whereas displays of fear are often blended with surprise (Ekman et al. 1987), and such blends are consistent across cultures (Ekman et al. 1987, p. 715). Notably, confusions between emotion categories which are closely related carry important information and meaningfully distinguish emotions among their interrelated emotion gradients (Cowen and Keltner 2020, 2021). Because of these shared features of emotion and because emotion displays may convey more than one emotion, we take the perspective that there are degrees of correctness for every potential emotion label rather than a single correct answer. We apply a theoretically appropriate Graded Response Model (GRM; Samejima 1969), which we elaborate on in Study 1. 

### 1.4. Concealed Emotions

Prior research has investigated the ability to eavesdrop on micro-expressions (Blanck et al. 1981; Elfenbein and Ambady 2002a; Puccinelli and Tickle-Degnen 2004); what is less understood, however, is how well people can pick up on *consciously* hidden emotions—which we refer to as *concealed emotions* (see also emotion masking: Ekman et al. 1990; Porter and Ten Brinke 2008). Scholars have noted that regulating one’s emotion expressions to suit a desired state or social situation constitutes a form of ‘emotion labor’ (Grandey and Melloy 2017; Hochschild 1983). As such, the ability to conceal one emotion with another might serve important social functions (e.g., parents mask their negative emotions with neutral or positive expressions to avoid upsetting their young children, though this may ultimately backfire: Le and Impett 2016; Waters et al. 2020). 

Research on the conscious concealment of emotions has found that observers can, at the very least, detect when an expression is authentic or not (i.e., deception)—although seemingly not with a high degree of accuracy (observers only correctly identify that a deceptive facial expression has occurred around 59% of the time; Porter and Ten Brinke 2008). As such, this research indicates that emotional concealment can be signaled by specific deception cues, such as excessive blinking (Porter and Ten Brinke 2008). However, prior work largely does not address whether or not perceivers can accurately label what emotion is being concealed. Initial work in this area suggests that detection of concealed emotions is difficult, with perceivers detecting the specific concealed emotion less than 20% of the time even among close friends (Sternglanz and DePaulo 2004). In this study, to advance the field we also examine the extent to which individuals can detect emotional states purposefully concealed by other emotional expressions meant to mask their true (underlying) emotion, and whether this perception ability is positively or negatively related to healthy social-emotional functioning.

### 1.5. Reducing Bias in Emotion Perception Assessments

Prior research has demonstrated that emotion perception accuracy is influenced by in-group advantage (Dovidio and Gluszek 2012; Elfenbein and Ambady 2002a; Laukka and Elfenbein 2021). Perceivers are quicker and more accurate at labeling emotions when the expressor belongs to the same social group, and they are slower and less accurate at labeling emotions when the expressor belongs to a different social group. There is both an in-group advantage as well as an out-group disadvantage. Groups can be defined and delineated by a range of factors, including demographic characteristics. One pattern is that the race of the expressor and perceiver may interact to affect response latency and accuracy (Bijlstra et al. 2010; Gitter et al. 1972; Hugenberg 2005; Young et al. 2012). When expressor and perceiver are the same race, there is an advantage that reduces response latency and increases labeling accuracy, whereas when expressor and perceiver are of different races, response times increase and accuracy decreases. This pattern also holds true across majority and minority groups in general, and findings show that majority group members are significantly worse at judging the expressions of minoritized group members (Elfenbein and Ambady 2002a). Additionally, racial minorities may be particularly susceptible to the potential harms of these biases with findings showing, for example, that teachers consistently misperceive the expressions of black children to be angrier than those of white children (Halberstadt et al. 2020). This research points to the importance of socio-cognitive accounts of racial bias in emotion perception where scholars have discussed the potential importance of in-group and out-group social dynamics that might motivate emotion misperception. This includes the potential that majority groups may simply disregard emotion expressions of outgroup members and instead process stereotypic information about a social outgroup (Young et al. 2012). 

Racial in-group advantage presents a substantial unaddressed challenge to measuring emotion perception. Prior assessments of emotion perception have typically focused on either a single race group (e.g., only using White expressors: Mayer 2002; Schlegel et al. 2014) or a comparison of two groups (e.g., comparing Asian and White expressors; Matsumoto et al. 2000). Research suggests that measures only containing White stimuli, for example, may unfairly advantage White perceivers through an own-race-bias advantage (Elfenbein and Ambady 2003; Young et al. 2012). Given the important role race plays in the accuracy of perceiving emotions, in this study we use a racially diverse group of White, Black, Hispanic, and Asian expressors. 

Furthermore, prior research also demonstrates that gender is an important demographic moderator for emotion perception ability. Across studies, women tend to outperform men on emotion perception ability (Brackett et al. 2004; Brody and Hall 2008; Joseph and Newman 2010; Olderbak et al. 2019). Therefore, in this study we also examine emotion expression across gender and use a balanced number of male and female expressors.

To assess the extent to which group advantages may exist in the MET for perceivers, we apply a measurement equivalence approach. Measurement equivalence quantifies the extent to which a test measures the same construct in the same way across sub-populations (LaPalme et al. 2016; Vandenberg and Lance 2000). Finding measurement equivalence across racial and gender sub-groups is important because tests are only fair when scores have the *same meaning* across target populations. If scores cannot be equated across sub-groups, this threatens the validity of score interpretation (American Educational Research Association et al. 2014) and of the construct of emotion perception itself. Moreover, as emotion skills become of increasing interest in business and education as targets of assessment and development—it is important to develop and use race and gender fair measures to inform decision making (Council of National Psychological Association for the Advancement of Ethnic Minority Interests 2016). Accordingly, in our creation of the MET, we validated racially and gender-balanced stimuli on a racially and gender-balanced sample to permit the testing of measurement equivalence. 

### 1.6. Reducing Methodological Bias

In this section, we discuss and address the use of forced-choice labeling as a methodological bias that may undermine the rigorous study of emotion perception. Scholars have debated the use of paradigms where one must choose an emotion label from a short list of options as compared to freely labeling what they perceive (Barrett 2017a; Gendron et al. 2018). The primary critiques of the forced-choice paradigm are that it inflates the accuracy of emotion perception by providing the perceiver with the emotion concepts necessary to label the emotion, and by enabling more accurate emotion labeling through educated guessing between choices—issues which would not be present in an open-ended response format. In particular, the use of forced-choice paradigms may make correct answers more obvious when incorrect options are implausible. For example, a cackling display of amusement may be unlikely to be confused with anger, sadness, or boredom. Asking someone to answer by choosing among these labels, “amusement”, “anger”, “sadness”, or “boredom”, may make it more apparent that the answer is amusement as compared to a free-labeling approach. 

In this research, consistent with a fuzzy set view of emotion, we present emotion labels as a set of interrelated emotion concepts, and we purposely select distractor responses that vary on correctness and are plausible alternative responses. For example, a display of amusement might be paired with the plausible positive emotion distractors of joy, pride, and content. We based the plausibility of distractor items in our test not only on the documented and shared characteristics of emotions (Campos et al. 2013; Cowen and Keltner 2017, 2020, 2021; Shaver et al. 1987), but also on data indicating endorsements for these emotion labels as plausible (using a ‘confusion matrix’ statistical methodology, explained in Study 1 below). Accordingly, in the MET, we examine the extent to which individuals can distinguish among a diverse array of emotion expressions using an interrelated but distinct set of emotion concepts as labels for those expressions. Taken together, presenting multiple related emotion concepts does not inflate accuracy, but rather challenges individuals to choose the *most correct* label amidst a suite of options where discernment requires high levels of emotion perception ability. 

## 2. The Present Research

Across four studies, we develop, refine, and validate a new measure of emotion perception ability, called the MET. In Study 1, we develop the raw emotion stimuli from which our test is built and demonstrate that these emotional expressions are reliably recognized in a large race- and gender-balanced U.S. sample. In Study 2, we select a subset of our emotion stimuli and develop our measure of emotion perception by demonstrating its reliability, unidimensionality, and measurement equivalence (across race and gender). In Study 3, we show that the MET demonstrates convergent validity with prior validated measures of emotion perception ability and divergent validity from measures of cognitive ability. Finally, in Study 4, we report the predictive validity of the MET, specifically the MET’s relationship to a range of healthy life outcomes, including greater psychological well-being and empathic concern, caring interpersonal relationships, and higher self-awareness of thoughts and feelings, while showing that it is inversely related to depression, anxiety, and stress. We describe the rationale, methodologies, and results from all four studies below. The studies in this paper were not pre-registered. Data, syntax, and materials for all studies are available at this OSF link: (URL accessed on 1 July 2023 https://osf.io/7czyp/?view_only=ba134383e47d4bdbb5da2da40298d5a5).

## 3. Study 1

In Study 1, we generated novel, ecologically valid meso-expression stimuli to be used in the development of the MET (Study 2) using a racially and gender-balanced^2^ group of actors. 

### 3.1. Methods

#### 3.1.1. Expressers/Actors

Professional actors were recruited both from local drama departments and online advertisements on social media. Actor applicants who responded to our call were emailed an autobiographical recall prompt to recall a time they experienced awe, fear, sadness, or sympathy (emotions included in our study). Applicants were then allowed to submit short sample clips of the displays they produced via method acting to the researchers. We note that the autobiographical recall procedure we used is not only a validated mood induction in the literature, but is also a common method acting exercise in the Stanislavsky Method (which nearly all actors would be familiar with). We included two male and two female Asian actors, two male and two female Black actors, two male and two female Hispanic/Latinx actors, and two male and two female White actors. All actors were between the ages of 20 and 40. 

#### 3.1.2. Emotion Induction

Consistent with prior research using autobiographical recall emotion induction techniques (Coan and Allen 2007; Joseph et al. 2020; Salovey 1992) and with method acting (i.e., Stanislavski 1989), actors were asked to recall in vivid detail a time they felt each emotion. Prior to the filming, each actor received a pre-filming instruction packet that described the themes of each of the emotions (amusement, anger, anxiety, awe, boredom, contempt, content, disgust, embarrassment, fear, joy, pride, relief, sadness, shame, surprise, sympathy; see Supplemental Method and Results). They were then asked to recall two examples of times when they felt each of the emotions, including a time they felt a small amount of the emotion and a time they felt a moderate amount of the emotion. The actors were asked to follow an autobiographical emotion induction prompt adapted from Salovey (1992), which can be found in the Supplemental Methods and Results (URL accessed on 1 July 2023 https://osf.io/7czyp/?view_only=ba134383e47d4bdbb5da2da40298d5a5). 

In addition to each of the 17 emotions, actors were also asked to recall times when they had to conceal their emotions. Actors were prompted in the pre-filming packet to recall times they felt anger but displayed contentedness, felt contented but displayed anger, felt sadness but displayed joy, and felt joy but displayed sadness (we refer to these as ‘concealed’ emotion stimuli). We specifically selected these patterns of concealed emotions because: (1) prior studies have noted that people often try to mask an emotion with one of opposing valence (e.g., a parent concealing negative emotions with positive emotions: Le and Impett 2016), so these patterns are consistent with how emotion concealment occurs in real life; and (2) prior research suggests that when the concealed emotions are incongruent in valence and arousal, it causes more emotional leakage (Porter and Ten Brinke 2008), which increases the signal of the concealed emotion and the opportunity for perceivers to pick up on it. 

#### 3.1.3. Recording and Technical Procedures

Actors were filmed using three high-definition studio cameras and the audio was recorded using an overhead boom microphone. Actors were instructed to review the instruction packet before the filming to ensure their emotion-laden stories would be readily recalled during the filming. During the filming, the director instructed each actor to recall the emotional experiences before each emotion display using a method acting prompt (see Supplemental Method and Results). 

For each emotion, the actor was asked to give an emotion display both without using words and while speaking a predetermined sentence. Nonverbal displays included facial expressions, body movements, and nonverbal utterances (e.g., sighing for boredom). Verbal displays included vocal tone in the statement/question, “Hello. How are you?”. For the concealed emotion displays, actors were asked to recall times they felt anger but displayed contentedness, felt contented but displayed anger, felt sadness but displayed joy, and felt joy but displayed sadness. Finally, we asked each actor to give a neutral display (where no emotion was felt) because prior research on emotion perception has advocated for including neutral as an option when determining whether emotion stimuli are recognized above chance (Bänziger et al. 2012; Laukka et al. 2016), as it helps to prevent forcing perceivers to choose an emotion label when they believe none is expressed. All affective information conveyed by expressors was the result of their natural expressions in response to the prompts provided—no specific instructions about the ‘correct’ way to convey each emotion—whether through facial expressions, body movements, and/or nonverbal utterances—were given. This approach afforded us with more naturalistic, ecologically valid stimuli while retaining emotion specificity. 

#### 3.1.4. Validation of Stimuli

Using the procedures described above, we produced 914 unique displays (504 non-verbal displays, 313 verbal displays, and 97 concealed emotion displays). Each display lasted approximately 5–7 s. To determine the quality of these displays and to reduce the total number of stimuli to a more manageable set, we conducted a validation study (described below). 

#### 3.1.5. Participants 

Three thousand participants were recruited using an online panel. Because we were interested in ensuring that our racially diverse stimuli were validated in a fair manner, we used a racially diverse set of perceivers. We aimed to sample 750 Caucasian, 750 African-American, 750 Asian, and 750 Hispanic participants. Participants were citizens or permanent residents of the United States, 18 years of age or older, and fluent in English. Our final sample contained 1025 Asian (512 males and 513 female), 855 Black (342 male and 513 female), 840 Hispanic (327 male 513 female), and 1025 White (512 male, 513 female) participants for a total of 3745 participants. The average age of participants was 44.39 years old (SD = 13.93). Data were collected in the Fall of 2018 through the Spring of 2019.

#### 3.1.6. Procedures 

Participants were randomly assigned 32 stimuli to rate (16 non-verbal stimuli, 12 verbal stimuli, and four concealed emotion stimuli). Randomization occurred within stimuli type and within racial group of the expresser and actor.^3^ This was done to ensure that stimuli from expressers of each racial group were judged by perceivers of each racial group (e.g., Hispanic/Latinx stimuli were rated by Asian, Black, Hispanic/Latinx, and White perceivers). The randomly selected stimuli were also presented in randomized order to reduce the possibility of order effects. Finally, participants were prompted to answer four attention check questions at random intervals during the survey. 

#### 3.1.7. Ratings 

After viewing each stimulus, participants provided several ratings. Participants first indicated which of the emotions the actor was trying to express using a multiple-choice response format. Each question had 17 potential emotion labels and the option to select neutral, and only one option was allowed to be selected. The inclusion of both neutral stimuli and the option to label a display as neutral helps to avoid forcing participants to label an emotion when they believe none is present. Additionally, by providing a wide range of possible answers, some of which are similar emotion concepts, we reduce the risk of answers resulting from the process of elimination. Participants then indicated how intense the expression was on a five-point scale from 1 (very slight) to 5 (very strong). Finally, participants indicated how authentic the emotion expression was on a five-point scale from 1 (not at all believable) to 5 (very believable). These measures served as our manipulation checks to ensure that our stimuli were in fact meso-expressions (i.e., moderate in intensity) and that they were perceived as believable expressions (rather than contrived or posed displays). 

For concealed emotion displays, participants were first asked to indicate what emotion the actor was trying to express and then they were asked to indicate which emotion the actor was trying to hide. Both questions presented all 17 emotions (plus neutral stimuli) in a multiple-choice response format, and only one option was allowed to be selected for each question. Afterward, participants were asked to rate the intensity of the expression and the believability of the expression using the same five-point scales described above. The concealed emotion displays were presented in a separate trial.

#### 3.1.8. Analyses

Using multiple choice responses, we calculated the proportion of participants who selected the intended target emotion for each stimulus (hereafter referred to as the *hit rate*). Across emotions, we also calculated a confusion matrix (see Table 1 and Table 2). Confusion matrices are a widely used test development tool that have been applied in previous emotion test development research (Laukka et al. 2016). Primarily, a confusion matrix shows the proportion of respondents who choose the intended emotion on the diagonal of the matrix (i.e., how often the target emotion was recognized), and the proportion of respondents that choose any of the candidate ‘distractors’ on the off-diagonal. 

To make our hit rates easier to understand, we converted all raw hit rates to a proportion index based on the total number of response options (i.e., 17 emotions plus neutral). This proportion index (pi; Hall et al. 2009; Rosenthal and Rubin 1989) represents our hit rates as if the judgement was made dichotomously; thus, the chance level of accuracy is 0.50. 

The proportion index is calculated as:

$$pi=\frac{P(k-1)}{1+P(k-2)}$$

where *P* is the observed hit rate and *k* is the number of response options (i.e., 18). The advantage of using *pi* is that our hit rates become comparable across judgements with differing number of options (e.g., our study can easily be compared to other interpersonal judgement studies: Hall et al. 2008; Juslin and Laukka 2003), and it makes determining if a stimuli was recognized above chance simple (i.e., any stimuli recognized more than *pi* = 0.50, or more than 50% of the time, is considered above chance).

We did not correct hit rates based on response biases.^4^ Finally, for each stimulus, we calculated the mean perceived intensity of the expression and mean perceived believability of the expression. 

### 3.2. Results 

For both the nonverbal and verbal stimuli, on average, all emotion categories were recognized above chance (see Table 1 and Table 2). This indicates that, on average, perceivers could accurately determine the intended expressed emotion. For the nonverbal stimuli, the mean *pi* (i.e., the average hit rate across all 504 non-verbal stimuli) was 0.81 (*SD* = 0.18), while for the verbal stimuli the mean *pi* was 0.59 (*SD* = 0.25). Additionally, perceivers rated stimuli, on average, as being both moderate in intensity and believability (note ratings used a five-point scale). For nonverbal displays, the perceiver-rated mean intensity was 2.74 (*SD* = 0.27) and the mean believability was 3.14 (*SD* = 0.24). For verbal displays, the perceiver-rated mean intensity across emotions was 2.64 (*SD* = 0.19) and the mean believability was 3.06 (*SD* = 0.16).

For the concealed emotion stimuli, recall that perceivers had to label both the emotion that was expressed and the emotion that was concealed. For these stimuli, we considered a ‘hit’ to be when a perceiver correctly labeled both the expressed and concealed emotion. The mean *pi* (i.e., the average hit rate across all 97 concealed stimuli) was 0.54 (*SD* = 0.41). Mean hit rates were lower for the concealed stimuli because there were 324 total potential unique responses (i.e., 18 potential expressed emotions multiplied by 18 potential concealed emotions). Perceivers rated these stimuli as both moderate in intensity (*M* = 2.73, *SD* = 0.14), and as expected for these concealed displays, low in believability (*M* = 1.05, *SD* = 0.06).^5^ For the concealed stimuli, we present confusion matrices for expressed and concealed emotions in Table 3. 

### 3.3. Discussion 

**Figure 1:**
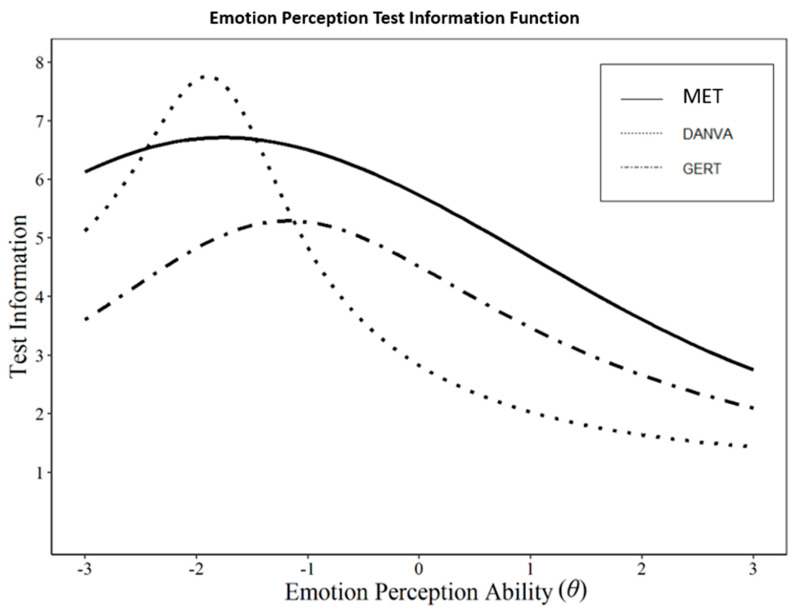
Test Information Function.

This study demonstrated that it is possible to elicit and reliably measure nuanced, highly variable, and ecologically valid meso-expressions (as opposed to the dominant paradigms of macro-expressions and micro-expressions). Actors in our study recalled their own emotion circumstances, and produced spontaneous and authentic displays of emotion (with no coaching on which facial muscles to activate). We found consistent evidence that our method acting autobiographical recall emotion manipulation achieved the goal of eliciting meso-displays with moderate intensity (the mean intensity rating was 2.64 out of 5) that were, on the whole, considered believable (the mean believability rating was 3.14 out of 5).^6^ Furthermore, we found that these emotion expressions were consistently recognizable to a broad array of perceivers varying on race/ethnicity and gender. Moreover, we found additional evidence that emotion categories are a fuzzy set because participants consistently choose plausible distractors for expressed emotions. That is, answer patterns to emotion stimuli were clustered non-randomly based on degrees of shared features among emotion concepts (e.g., phenomenology, appraisals, action tendencies). For example, in Table 1, we find that fear is consistently confused with anxiety (another high-activation negative emotion), while sadness is consistently confused with shame (another low-activation negative emotion: Cowen and Keltner 2017, 2020, 2021; Figure 1). Employing stimuli developed in Study 1, we next construct a novel assessment of emotion perception ability in Study 2 called the MET.

## 4. Study 2

In Study 2, we developed the Meso-Expression Test (MET) using a subset of the stimuli we validated in Study 1. To develop this test, we included displays of amusement, anger, boredom, contentment, disgust, embarrassment, fear, joy, relief, sadness, shame, surprise, and sympathy. We selected stimuli based on the quality of the display and only included displays that were recognized above chance in Study 1 to ensure they were valid emotion displays. For each given emotion, we selected at least one stimulus from each racial and gender subgroup that was recognized above chance, but that was also rated as moderately intense and at least moderately believable (see Study 1) to ensure it captured a meso-expression. As a result of this process, among the candidate stimuli, there was at least one male and one female expresser of each race (e.g., Asian, Black, Hispanic, and White) for each emotion. We selected a total of 104 non-verbal displays and 44 verbal displays to test. 

Finally, for the concealed emotion stimuli, we selected displays where expressers felt anger but displayed contentment, felt contentment but displayed anger, felt joy but displayed sadness, and felt sadness but displayed joy. We specifically selected 20 stimuli and emphasized selecting displays that were recognized above chance in Study 1 and that were moderate in intensity (noting that believability was not a factor in selecting these stimuli, as concealed emotion displays appear less natural).

Thus, Study 2 focuses on the 168 stimuli above that we develop into a candidate item pool. In this study, we psychometrically validate these candidate items and cull items that do not meet our rigorous psychometrics standards outlined below. 

### 4.1. Methods

#### 4.1.1. Participants

We aimed to recruit a total of 4000 participants using an online panel. Participants were employed permanent residents or citizens of the United States who were 25 years of age or older and were fluent in English. Furthermore, because we were interested in ensuring that our race- and gender-balanced stimuli were validated in a fair manner, we sampled a race- and gender-diverse set of perceivers. Using a purposeful sample balanced on race and gender allowed us to test for measurement equivalence across groups (described below). 

Because of the large number of stimuli we needed to validate, we chose to examine each stimuli type (non-verbal, verbal, and concealed emotions) in separate sub-samples. Thus, Study 2 consisted of three sub-samples, each corresponding to a separate sub-test (nonverbal, verbal, and concealed emotion perception). Our sample consisted of 1598 participants balanced on race and gender for the nonverbal stimuli (802 women, 796 men, 399 Asian, 401 Black, 397 Hispanic, and 397 White participants; mean age was 47.15 *SD* = 14.11), 1196 participants balanced on race and gender for the verbal stimuli (598 women, 598 men, 299 Asian, 300 Black, 297 Hispanic, and 300 White participants; mean age was 44.39 *SD* = 13.93), and 1195 participants balanced on race and gender for the concealed stimuli (600 women, 595 men, 296 Asian, 299 Black, 300 Hispanic, and 300 White participants; mean age was 44.48 *SD* = 14.10). Data were collected in the Spring of 2019.

#### 4.1.2. Selection of Distractor Items

Distractor response options are an important part of test development and the quality of distractors selected determines the difficulty and discrimination of items (Andrich and Styles 2011; Rodriguez 2005). We chose to select distractors at the item level based on the confusions of emotion labels from Study 1. We present the details of this method and the item-level confusion matrices (Tables S1 and S2) in a Supplemental Methods and Results (URL accessed 1 July 2023 https://osf.io/7czyp/?view_only=ba134383e47d4bdbb5da2da40298d5a5). For the verbal and nonverbal sub-tests, we retained the most correct answer and five additional distractors; participants were asked to select one answer. For each item, at least one of the distractors was a partially correct answer (i.e., it shared emotion concept features with the target emotion and had high rates of confusion with the target emotion; see Table S1). Any remaining answers were also plausible incorrect distractors (i.e., emotion labels that may be confused with the target emotion and may still share some common features with the target emotion). 

The concealed emotion sub-test was designed such that participants had to discern both the expressed emotion and the emotion that was actually felt by the expressor (i.e., the concealed emotion). Compared to the nonverbal and verbal sub-tests, the concealed emotion sub-test was more difficult because it required perceivers to understand both the external and concealed internal states of the expresser. For this emotion sub-test, both the expressed emotion and the concealed emotion were paired with four distractor responses each; participants were asked to select one answer for each question. At least one of the distractors was a plausible partially correct answer (i.e., it had high rates of confusion with the target emotion and shared features with the target emotion), and any remaining answers were plausibly incorrect distractors (based on overlap in emotion concepts and families).

In Table S2, we show the item-level results from Study 2. Across items, we replicate results from Study 1 and find that correct emotion labels were all selected above chance. Additionally, we find that partially correct distractors were selected frequently and, in most cases, more so than the plausible incorrect distractors as expected. 

#### 4.1.3. IRT Scoring

We also chose to employ a Graded Response Model (GRM; Samejima 1969), given that many emotions share conceptual and phenomenological similarities (e.g., Cowen and Keltner 2017, 2020, 2021; Shaver et al. 1987). Given that many emotions may be confused based on their shared conceptual and phenomenological qualities, a GRM is appropriate because all item responses (the best response and the distractors) may be ordered in terms of their degree of correctness. For the verbal and nonverbal sub-tests, item responses were scored trichotomously as: 0 (inaccurate), 1 (partially correct), or 2 (correct). Responses were scored as *correct* when the target emotion was selected, *partially correct* when a plausible distractor was selected, and *incorrect* otherwise. Prior cognitive ability research has used GRM approaches to effectively measure gradients in latent mental abilities (Sternberg et al. 2014), but it appears the MET is the first assessment of emotion perception ability to apply this methodological technique. 

As each concealed display required two judgements, we scored these items using both the judgement of felt and expressed emotion simultaneously. For the GRM, item responses were scored as: 0 (inaccurate), 1 (somewhat correct), 2 (mostly correct), and 3 (correct). Responses were scored as *correct* when the perceiver selected both the felt and expressed emotion, *mostly correct* when the perceiver selected the correct emotion labels but confused the felt and expressed emotion (e.g., selected felt anger and expressed contentment, when the expresser actually felt contentment and expressed anger), *somewhat correct* when only one correct emotion label was selected, and *incorrect* for all other responses. 

#### 4.1.4. Measurement Equivalence

One goal of including a racially diverse set of *expressers* in the test was to increase its fairness across racially diverse groups of *perceivers*. In order to quantify the fairness of the test across different racial groups of perceivers, we conducted measurement equivalence (ME) analyses. ME examines the extent to which a test measures the same construct in the same way across groups (Vandenberg and Lance 2000). ME testing helps to ensure that differences in responses can be interpreted in a meaningful and unbiased manner rather than being confounded by group membership (LaPalme et al. 2016). In this case, it means ensuring that differences in scores on the MET primarily reflect emotion perception ability, and largely do not reflect the race or gender of the test taker. We also were interested in ensuring ME across male and female test takers because prior research has shown that women outperform men on EI ability tasks (Joseph and Newman 2010).

We used a Differential Item Functioning (DIF) approach to examine ME. The advantage of a DIF approach (as compared to more traditional ME analyses) is that DIF examines measurement equivalence at the item level rather than at the scale level. That is, DIF can identify which items are not equivalent across groups, and makes it possible to determine if specific items are not equivalent across groups (even when the total scale appears equivalent). Additionally, DIF makes it easy to quantify the effect size of non-equivalence between groups (i.e., how large group differences are). In this study, we operationalized DIF as the squared area between the ICCs of the comparison groups divided by the pooled standard deviation (LaPalme et al. 2016; Nye 2011). We compared the ME of our test for Asian versus Hispanic, Black versus Hispanic, Black versus Asian, White versus Black, White versus Hispanic, White versus Asian, and men versus women. 

#### 4.1.5. Results

We examined multiple indicators of item quality and item functioning, and we present evidence for each sub-test below. 

#### 4.1.6. Nonverbal Displays

For the nonverbal sub-test, the average factor loading across all 104 candidate items was 0.27 (*SD* = 0.12), demonstrating moderate average factor loadings and the need to potentially cull items with low loadings. We also examined item information. Item information represents the precision of measurement provided by an item, which is inversely related to the standard error of measurement across the latent continuum of emotion perception ability. Item information is important to examine because items that provide little information do not improve the precision of tests, whereas items with high information values lead to less measurement error (Lord 2012).^7^ Higher item information values are preferred to lower item information values, so we can compare which items perform better relative to the mean information value across items. The mean candidate item information was 0.40 (*SD* = 0.28).^8^ Finally, we also examined item misfit. Item misfit indicates that responses to an item did not fit the expected or modeled responses well. Out of the 104 candidate items, 19 items had statistically significant misfit. To further examine misfit, we also computed the chi-square to degrees-of-freedom ratio.^9^ Ratios greater than or equal to three are interpreted as poor fit, while ratios that are less than three are interpreted as adequate fit (Tay et al. 2011); all the items examined had adequate fit using this standard. 

We chose to cull nonverbal candidate items that had a combination of: (1) significant misfit, (2) poor factor loadings (i.e., loadings below 0.30), and/or (3) provided below average item information. As such, we removed 54 items (which had poor fit, poor factor loadings, and/or low item information) and retained 50 items. From this set of 50 retained items, to ensure content validity of the MET, we selected 31 items such that there were displays to represent both high activation and low activation positive and negative emotions (Russell 1980), and such that each emotion quadrant had male and female expressors, as well as expressors of different racial backgrounds. Table 4 displays the item statistics for the retained items. The average factor loading across the 31 retained items was 0.41 (*SD* = 0.12) and the average item information was 0.75 (*SD* = 0.28), which indicates measurement precision improved after culling items. The internal consistency reliability of the 31-item nonverbal test was *α* = 0.81 and McDonald’s Omega was 0.81. 

#### 4.1.7. Verbal Displays

For the 44 verbal candidate items, the average factor loading was 0.28 (*SD* = 0.12). The mean candidate item information was 0.44 (*SD* = 0.32). Finally, we also examined item misfit. Out of the 44 candidate items, 10 items had significant misfit. To further examine misfit, we also computed the chi-square to degrees-of-freedom ratio. All items had ratios less than three, which indicates adequate fit.

We culled 24 items (including all relief items, which had poor factor loadings and low item information) and retained 20 items. Table 5 displays the item statistics for the retained items. The average factor loading across the 20 retained verbal sub-test items was 0.37 (*SD* = 0.08) and the average item information was 0.68 (*SD* = 0.27), suggesting improved measurement precision. The internal consistency reliability of the 20-item verbal sub-test was *α* = 0.71 and McDonald’s Omega was 0.71.

#### 4.1.8. Concealed Displays

For the concealed emotion sub-test, the average factor loading across all 20 candidate items was 0.36 (*SD* = 0.13). The mean candidate item information was 0.80 (*SD* = 0.51). Out of the 20 items, 12 items had significant misfit. To further examine misfit, we also computed the chi-square to degrees-of-freedom ratio. All items had ratios less than three, which indicates adequate fit.

We culled seven items and retained 13. Table 6 displays the item statistics for the retained items. The average factor loading across the 13 retained items was 0.45 (*SD* = 0.08) and the average item information was 1.10 (*SD* = 0.40). The internal consistency reliability of the 13-item concealed emotion sub-test was *α* = 0.71 and McDonald’s Omega was 0.71. 

#### 4.1.9. Measurement Equivalence

Finally, we found that the test had only small differences across racial and gender groups in our ME analysis, which indicates that the MET fairly assesses emotion perception ability across race and gender. Effect sizes for DIF analyses can be interpreted similarly to a Cohen’s *d* (LaPalme et al. 2016; Wang et al. 2013), where 0.2, 0.5, and 0.8 represent small, medium, and large effect sizes, respectively (Cohen 1988). All effect sizes are reported in Table 7. For the nonverbal sub-test and concealed emotion sub-test, the average *d*-DIF was small across all race comparisons, and also across men and women. For the verbal test, the average *d*-DIF was also small across race comparisons, and it was small to moderate for the White versus Asian comparison, and also small across men and women. No items examined had large DIF between groups. 

### 4.2. Discussion

In Study 2, we developed a novel measure of emotion perception ability called the MET assessment. We employed naturalistic, ecologically valid meso-expressions in a racially and gender-balanced sample, where we found evidence for the internal consistency and structural validity of the MET. Additionally, across each of the sub-tests (i.e., nonverbal, verbal, and concealed emotion tests), we found moderate to high reliability of the emotion stimuli. We also showed that the MET test, as designed, largely operated equally across gender and four racial groups in the U.S., meaning there were only small differences in performance on the measure on the basis of race and gender. The MET is the first emotion perception measure to examine and demonstrate overall test fairness across racial and gender groups. A sample of the assessment may be found here: (URL accessed 1 July 2023 https://osf.io/7czyp/?view_only=ba134383e47d4bdbb5da2da40298d5a5).

## 5. Study 3

In Study 3, we examined the construct validity (convergent and divergent validity) of all three subtests of emotion perception on the MET. Our goals were to test whether: (1) the MET converges with other measures of emotion perception ability, and (2) the MET is distinct from cognitive ability.

### 5.1. Methods

#### 5.1.1. Participants

Two hundred and eighteen participants were recruited from a university in the Northeastern United States. The participants were 71.1% female, and were 16.5% African-American, 36.2% Asian, 9.2% Hispanic, 0.5% Native American, 33.6% White, and 3.7% identified as other. The mean age of the sample was 24.81 years old (*SD* = 10.55). Data were collected in the spring of 2019. 

#### 5.1.2. Measures

We used a 64-item version of the MET (31 nonverbal items, 20 verbal items, and 12 concealed items). In addition to the MET, we included measures of convergent and divergent validity, which are two core components of construct validity (Cronbach and Meehl 1955). 

To demonstrate convergent validity, we included two previously validated measures of emotion perception: the Diagnostic Analysis of Nonverbal Accuracy (DANVA; Nowicki and Duke 1994) and the short Geneva Emotion Recognition Test (GERT-S; Schlegel and Scherer 2016). For the DANVA, we used the 23-item face recognition and 23-item nonverbal recognition portions of the test. These examine the ability to recognize happiness, sadness, anger, and fear via facial expression and through non-verbal vocal cues, respectively. The GERT-S consisted of 42 short videotaped emotional expressions. These emotional expressions contained both bodily expression of emotion and nonverbal emotion expression. Perceivers were asked to label the emotion expressions as amusement, anger, disgust, despair, pride, anxiety, interest, irritation, fear, pleasure, relief, surprise, or sadness. Both the DANVA and GERT items were scored as either: correct (1) or incorrect (0) using a two-parameter logistic Rasch model (Rasch 1960). 

We tested divergent validity using the 30-item quick form of the Wonderlic Personnel Test (WPT-Q; Wonderlic, Inc. 2007). The WPT-Q is a test of cognitive ability that examines the ability to learn, adapt, and solve problems. 

### 5.2. Results

Table 8 shows the correlations between the MET and our measures of convergent and divergent validity. The subtests of the MET (nonverbal, verbal, and concealed emotion perception) were highly correlated with one another (*r* = 0.48 to 0.59, *p* < .001); the concealed MET is correlated 0.48 with the nonverbal MET, 0.50 with the verbal MET, and 0.82 with the MET total score. The MET demonstrated convergent validity with both the DANVA (*r* = 0.59, *p* < .001) and the GERT (*r* = 0.68, *p* < .001). Finally, like other emotion perception tasks, the MET was significantly positively related to cognitive ability though the effect was moderate in size (*r* = 0.36, *p* < .001; Cohen 1988), supporting overall its divergent validity (Mayer et al. 2008). 

To demonstrate the incremental validity of the MET (beyond the other perception measures in this study), we also compared the total information provided by the test to the DANVA and the GERT. As discussed in Study 2, item information is inversely related to the standard error of measurement. Tests that provide more information are more precise, whereas tests with lower information have more measurement error. The more information a test provides consistently, the better the measure is tapping the construct of interest. Figure 1 shows the test information function (TIF) for the MET, DANVA, and GERT across the emotion perception ability continuum from (*θ* = −3 to +3). On average, the MET provided significantly more information than the DANVA, *t*(59) = 9.88, *d* = 2.57, *p* < .001, or the GERT, *t*(59) = 21.87, *d* = 5.69, *p* < .001. Further, the total information provided by the MET (i.e., the area under the TIF curve) was 31.8 compared to 22.4 for the DANVA and 23.7 for the GERT. Figure 1 also shows that the MET provides consistently high information value across low, moderate, and high perception ability levels. 

### 5.3. Discussion

In Study 3, the MET converged with two widely used measures of emotion perception ability (the DANVA and GERT), demonstrating that it taps the same emotion perception construct (convergent validity). We also found that the MET is largely distinct from cognitive ability, supporting its divergent validity (noting a moderate correlation is common between different mental ability tests; Mayer et al. 2008). Together, these findings support the construct validity of the MET, placing it in a nomological network where it is highly associated with other emotion perception tests and more weakly associated with cognitive ability tests (Cronbach and Meehl 1955). Finally, importantly, the MET also showed incremental value above and beyond the DANVA and the GERT in that it provides more test information about emotion perception ability across a wide range of the latent continuum (i.e., it is suitable for assessing emotion perception ability in low, medium, and high perception ability individuals). We note that a limitation of Study 3 is that the sample was majority female, and women outperform men on assessments of EI. In study 4, we use a gender-balanced sample to examine gender differences on the MET.

## 6. Study 4

In Study 4, we assessed the criterion-related validity of our emotion perception test on psychosocial outcomes. Based on prior studies relating emotion perception to psychosocial outcomes, in Study 4 we assessed the relationship between the MET with social well-being, empathic concern, social connectedness, relationship quality, alexithymia, stress, depression, and anxiety. We hypothesized that emotion perception measured by the MET would be positively associated with well-being, empathic concern, social connectedness, and relationship quality, and negatively related to alexithymia, stress, depression, and anxiety (e.g., Fernández-Berrocal and Extremera 2016; Mayer et al. 2008). We also investigated whether perceiving concealed emotions would be positively or negatively related to relationship quality since prior research on emotional eavesdropping has shown the potential for negative effects of detecting leaked concealed emotions on social relationships.

### 6.1. Methods

#### 6.1.1. Participants

We sampled 740 participants using an online panel. Participants were employed individuals in the United States 18 years of age or older and were selected to be racially/ethnically and gender-representative of the US workforce. The final sample of participants were 48.8% female, and were 10.5% African-American, 4.6% Asian, 67.2% Caucasian, 14.6% Hispanic, 0.9% Native American, and 0.8% Pacific Islander; 1.6% identified as other. The mean age of the sample was 39.36 years old (*SD* = 17.51). Data were collected in the summer of 2020.

#### 6.1.2. Measures

We examined the relationship of emotion perception ability with social well-being, empathic concern, social connectedness, relationship quality, alexithymia, stress, depression, and anxiety. 

Social well-being. For social well-being, we used the Ryff and Keyes (1995) Positive Relationships subscale of the Psychological Well-Being (PWB) scale. Scale reliability was α = 0.60. 

Empathic Concern. For empathic concern, we used the seven-item empathic concern sub-scale of the Interpersonal Reactivity Index (IRI; Davis 1980). Scale reliability was α = 0.58. 

Social connectedness. For social connectedness, we used the eight-item Lee and Robbins (1995) Social Connectedness Scale (CSC). Scale reliability was α = 0.94. 

Alexithymia. We measured alexithymia using the twenty-item Toronto Alexithymia Scale (Bagby et al. 1994). Scale reliability was α = 0.86. 

Stress. We measured stress using the Perceived Stress Scale (PSS; Cohen et al. 1994) which conceptualizes stress as the degree to which one appraises situations in one’s life as stressful. Scale reliability was α = 0.70.

Depression and anxiety. We measured depression and anxiety using the PROMIS Anxiety and Depression scales (Broderick et al. 2013; Schalet et al. 2016). Scale reliability was α = 0.91 and α = 0.92 for the anxiety and depression scales, respectively. 

Relationship quality. Finally, we measured the quality of close relationship, using the short version of Network of Relationships Inventory (NRI; Furman and Buhrmester 2010). This scale asked participants to think of a close significant other, and to answer questions about positive relationship factors such as social support (e.g., ‘How much does this person show support for your activities?’) and negative relationship factors (e.g., ‘How much do you and this person get on each other’s nerves?’). Scale reliability for positive relationships was α = 0.86, and α = 0.94 for negative relationships. 

### 6.2. Results

Correlations are reported in Table 9. The MET was significantly positively related to social well-being, empathic concern, and social connectedness, and significantly negatively related to alexithymia, stress, depression, anxiety, and negative interpersonal interactions with significant others. The MET also had a small unexpected negative relationship with positive social interactions with significant others. 

We also tested the concealed emotion scores separately to determine whether or not they were related to positive or negative outcomes. Concealed emotion perception was significantly positively related to empathic concern (*r* = 0.23, *p* < .001) and social connectedness (*r* = 0.17, *p* < .001), and significantly negatively related to alexithymia (*r* = −0.24, *p* < .001), depression (*r* = −0.19, *p* < .001), and anxiety (*r* = −0.20, *p* < .001). Additionally, the concealed emotion score was negatively correlated with negative interpersonal interactions with significant others (*r* = −0.34, *p* < .001), and as with the full test, had a small unexpected negative relationship with positive social interactions with significant others (*r* = −0.07 *p =* .048). Concealed emotion perception was not significantly related to social well-being (*r* = 0.06, *p.* = .10) or stress (*r* = 0.06, *p* = .20). 

#### Gender

Prior research has shown that mean EI scores (Cabello et al. 2016, p. 1486) and the relationship between EI constructs and psychosocial outcomes may differ between men and women (Brackett et al. 2004; Joseph and Newman 2010), and in our study, men underperformed on the MET compared to women (*r* = −0.30, *p* < .05). As such, we followed up by testing gender as a moderator. We entered gender and a gender x MET score interaction term into separate regressions for each outcome of interest and we report the interaction betas and significance below. Gender was a significant moderator of the relationship between emotion perception and empathic concern (*β* = −0.30, *p* < .05), stress (*β* = −0.51, *p* < .05), depression (*β* = −0.44, *p* < .05), anxiety (*β* = −0.38, *p* < .05), positive relationships (*β* = −0.34, *p* < .05), and negative relationships (*β* = −0.26, *p* < .05; see Table 10 for regression models). Gender was also a marginally significant moderator of social well-being (*β* = −0.26, *p* < .10), but was not a moderator for the social connectedness (*β* = −0.10, n.s.) or alexithymia associations (*β* = −0.12, n.s.). Following up these gender interactions, we report correlations for women and men separately in Table 11. The trend of the interactions and correlations show that women received more of the social well-being and empathy benefits of emotion perception ability, whereas men received lower stress and depression benefits of high emotion perception ability. One notable difference is that we found that MET scores were not related to social well-being for men (*r*= 0.06, n.s.) and had a small negative relationship with positive social interactions (*r*= −0.33, *p* < .05), which was not true for women and is consistent with prior research showing differences in the effects of emotion perception ability between men and women (Brody and Hall 2008; Joseph and Newman 2010; Olderbak et al. 2019).

### 6.3. Discussion

In Study 4, we set out to examine the initial criterion validity of the MET. We found that the MET was significantly associated with a number of healthy psychosocial and emotional outcomes identified in prior research. Across all participants, higher MET scores were associated with lower depression, stress, and anxiety, and negative interpersonal conflict as well as greater awareness of feelings (i.e., lower alexithymia and higher empathy). Consistent with prior findings in the emotion perception literature, MET correlations with psychosocial and affective indicators of healthy functioning were moderated by gender. For women, emotion perception ability was more strongly related to most indicators of social functioning and health, whereas for men we found that emotion perception may actually show small negative correlations with healthy relationships (e.g., a reduction in close positive social interactions). Women’s MET scores also did not correlate with stress, whereas men’s scores did.

## 7. General Discussion

In the present research, we investigate the existence, measurability, and psychosocial value of perceiving meso-expressions. Prior research has suggested that emotion perception is the primary ability of EI (Joseph and Newman 2010) and in this study, we advance the literature by defining meso-expressions as the dynamic, conscious moderate-intensity emotions of everyday life. Across four studies, we develop and validate a novel measure of emotion perception ability—the MET. There are multiple theoretical and practical contributions as well as implications of this research for affective science.

First, we found that meso-expression perception ability was correlated with an array of psychosocial outcomes and that the MET showed validity above and beyond other popular measures of EI. Across all participants, higher MET scores were associated with lower depression, stress, anxiety, and negative interpersonal conflict as well as greater awareness of feelings (i.e., lower alexithymia and higher empathy). These findings comport with prior meta-analytic evidence (Fernández-Berrocal and Extremera 2016; Sánchez-Álvarez et al. 2016; Mayer et al. 2008), and suggest that emotion perception ability reliably predicts a wide range of healthy and unhealthy psychosocial outcomes in a diverse pool of individuals. This is likely the case for a number of reasons, including that emotion perception plays a foundational role in facilitating effective emotion understanding and emotion regulation processes (Joseph and Newman 2010), and it plays a key role in the quality of social relationships, which are among the most robust predictors of psychological health and adjustment across the lifespan (Coan and Sbarra 2015; Pietromonaco et al. 2013; Sbarra and Coan 2018). We also show that the MET outperforms both the DANVA and the GERT and gives more information about emotion perception ability across the continuum of ability (see Figure 1) which speaks to the importance of the contribution of the MET. 

We note that, as with prior studies of EI, these findings were moderated by gender. We found that men underperformed on the MET compared to women, and that women received more of the social benefit of meso-expression perception than men, but received no benefit to their stress levels. Relatedly, we found an unexpected negative correlation with positive relationship interactions for men only. Future research should explore why emotion perception ability might be associated with men experiencing fewer positive interactions with close friends and significant others. Prior research has identified such differences in the effects of emotion perception between men and women (Brody and Hall 2008; Joseph and Newman 2010; Olderbak et al. 2019), but it is not clear what mechanism may account for this. For example, some prior research has indicated that emotion perception ability may also allow others to read threatening information from close partners which can hurt close relationships (Simpson et al. 2003) and the gender roles of men could potentially intensify these effects on close relationships. 

Second, our paper helps to address a key theoretical tension in the literature by bridging the gap between what emotions are theorized to be and how they are measured in the EI construct. The vast majority of the literature to date has either examined macro-expressions (intense, dramatized emotions) or micro-expressions (subtle and unconscious emotions) (Ekman 2003; Matsumoto and Hwang 2011, 2014). In particular, the literature is dominated by a basic emotion paradigm, which views emotions as fixed, biologically innate patterns of activation. This view narrowly defines and measures emotions through intense, stereotypic displays (Ekman 1992, 1997; cf. Barrett 2017a; Buck et al. 2017). We contribute to the emotion literature by examining an alternative to this macro-expression paradigm: the meso-expression emotion paradigm. We examine meso-expressions—the moderately intense emotions of everyday life. These patterns of activation are not fixed (as in the basic emotion paradigm), and the ability to accurately label them requires the nuance to process the natural idiosyncrasies of each expressor and the variation of the eliciting situation. 

Third, our research also addresses multiple central methodological limitations of measuring emotion perception as a facet of EI. Notably, prior work has critiqued the use of artificial emotion displays that hamper one’s ability to make judgements about the accuracy of emotion perception (Barrett 2017a; Buck et al. 2017). Using autobiographical recall (a well-established emotion induction and method acting technique; Joseph et al. 2020; Salovey 1992; Stanislavski 1989), we elicited lived emotions in the expressors in our stimuli to address this longstanding critique. In line with componential theories of emotions (Frijda 2007, 2008; Ellsworth and Scherer 2003; Dael et al. 2012; see also Shaver et al. 1987), and recent extensions of this theory (Cowen and Keltner 2021), our work suggests that there are many varied patterns of activation which may reliably signal the same emotions to perceivers. Our approach to inducing emotions provides stimuli that capture authentic complex emotions that are still reliably labeled. 

Fourth, we are the first paper to examine emotion labeling through a theoretically appropriate ‘graded’ response model (GRM; Samejima 1969). That is, we theorize and measure emotion labels from the perspective of their varying degrees of correctness based on shared phenomenology (Cowen and Keltner 2017, 2020, 2021; Shaver et al. 1987). Prior research has predominantly examined emotion labels as either correct or incorrect based on consensus (Ekman and Friesen 1974; Matsumoto and Ekman 1988; Nowicki and Duke 1994; Schlegel and Scherer 2016), thus introducing a false dichotomy into the emotion literature that does not theoretically align with emotions as a fuzzy set. Prior correct or incorrect dichotomy has served to reinforce the view that emotions are ‘basic’ or discrete constructs because each emotion label must belong to a distinct ‘emotion fingerprint’ (Barrett 2006, 2017a, 2017b). Rather, the results of our study suggest that, in line with our meso-expression paradigm and componential theory of emotions (Frijda 2007, 2008; Ellsworth and Scherer 2003; Dael et al. 2012; and well-supported extensions of this theory, see Cowen and Keltner 2021), emotions are a *fuzzy set*. This means that specific emotions share common features (e.g., phenomenology, expressions, appraisals), as their categories overlap with one another to varying degrees, so they are not mutually exclusive constructs, though they also carry unique variance. Our novel assessment of emotion perception advances the literature by conceptualizing emotions as a fuzzy set and applying a theoretically appropriate Graded Response Model.

We found consistent evidence of this important phenomenon in Table 1 and Table 2. Across the numerous emotion labels included in our study, *every* intended emotion expression yielded a *non-zero* chance of being labeled as another emotion category. Furthermore, emotion labels which had highly shared phenomenology and appraisals tended to be selected together. For example, anxious emotion expressions tended to be labeled above chance as anxiety or fear, demonstrating the fuzziness between these two high-activation, negative emotions. At the same time, however, there is a reliable signal that can be discerned and used to distinguish between emotion expressions (each expressed emotion was detected above chance, and above and beyond related emotion labels). Some prior work has viewed this fuzziness between emotion sets as ‘response bias’ (Lynn and Barrett 2014) and treated it as a source of measurement error (Elfenbein and Ambady 2003), which may unintentionally perpetuate the idea that emotions are discrete kinds. Our results clearly suggest that emotion labels are a fuzzy set and that there is a utility in conceptualizing and measuring the ‘confusions’ between emotion labels as partially correct responses. This affords us the opportunity to better capture and study the dynamic complexity and richness inherent in emotion perception phenomena as they occur in daily life.

Fifth, our paper builds on the robust literature demonstrating the in-group racial advantages in face and emotion perception (Elfenbein and Ambady 2002a; Gitter et al. 1972; Hugenberg 2005; Young et al. 2012). Prior measures of emotion perception have largely used expressors of a single racial/ethnic background (typically White) or compared expressors of two different national origins. This presents a serious threat to validity in the literature because performance on emotion perception measures will favor the racial in-group (Council of National Psychological Association for the Advancement of Ethnic Minority Interests 2016). We address this methodological limitation by explicitly balancing the race of expressors in our studies to include Asian, Black, and Hispanic expressors in addition to White expressors, and by validating the stimuli on an equally racially and gender-balanced sample. We find that the MET shows measurement equivalence across an array of racial/ethnic group comparisons. We also showed similar measurement equivalence across male and female groups.

Finally, our study contributes to an understanding of how well people can detect purposely concealed emotion states. In light of growing research suggesting that people often feel compelled to hide their true feelings and engage in emotional labor (Hochschild 1983; Grandey and Melloy 2017), our research demonstrates that people can detect purposefully concealed emotion states. This finding complements prior research on the perception of micro-expressions and emotion eavesdropping (which has examined the unconscious leaking of emotions; Elfenbein and Ambady 2002a). We extend these fields by showing that *conscious* concealment of emotions is a phenomenon that can be reliably perceived by others. Additionally, we found that the ability to detect concealed feelings is highly correlated with emotion perception ability in general, and that detecting concealed feelings may be a specific facet of EI—a finding which suggests that this facet might be incorporated into EI theory. Furthermore, importantly, unlike perceiving leaked micro-expressions, we found that the perception of purposefully concealed emotions is associated with adaptive psychosocial functioning. This helps to support prior theoretical conjectures that, because perceiving unconscious emotions is a form of emotion eavesdropping, it may have negative social consequences (Blanck et al. 1981; Puccinelli and Tickle-Degnen 2004), whereas perceiving the conscious and surreptitious concealment of emotions in our study was a beneficial emotion ability.

### 7.1. Limitations and Future Directions

A central limitation of our research and the MET is that it is mono-cultural. In this research, we examined a wide variety of emotion expressions across expressors and perceivers who varied in gender and race. However, all of the expressors and perceivers in our study resided in North America—the United States, specifically. Prior research has noted that cultural differences play an important role in emotion expressions and perceptions, and emotion expression and perception can and do vary by cultural group (Gendron et al. 2018; Matsumoto 1992, 2001; Tracy and Robins 2008b). Therefore, a key next step for emotion perception research will be to extend our meso-expression approach to examine emotion expression in other cultures. While a substantial literature suggests that emotion expressions are recognized across cultures (Cordaro et al. 2018, 2020; Cordaro 2014; Matsumoto 2001), most of these studies have examined macro-expressions. Future research should test whether or not more naturalistic, moderate-intensity meso-expression displays also show degrees of cultural universality. Furthermore, in this study, we examined only a small subset of gender (male and female) and racial (Asian, Black, Hispanic, and White) identities. Future research should consider examining emotion perception across a broader array of diverse expressers including non-binary expressors or indigenous expressors. 

Another limitation of this research is that our criterion validity test (Study 4) relied on cross-sectional self-report data. As such, the direction of association between emotion perception ability as indicated by the MET and the reported psychosocial functioning is unclear. Furthermore, the association between meso-expressions measured by the MET and social-behavioral outcomes in real social contexts is unknown. Future research should employ longitudinal designs and include measures of social ratings or actual social behavior to afford temporal separation between emotion perception and to measure the psychosocial outcomes of interest more objectively to determine the predictive validity of the MET. Furthermore, across our validation studies, we did not examine the test–retest reliability of the MET. Future research should examine the stability of MET scores across time using a test–retest paradigm to further demonstrate the reliability of the MET. 

One aspect of our study that improves the ecological validity of our stimuli is that we utilized autobiographical recall to elicit emotions from our actors. This means that emotions were reproduced rather than based on prototypical activation (e.g., asking someone to fake a smile). While there is evidence that autobiographical recall is a valid method of eliciting emotions, it still does not confirm that an actor actually felt that emotion. Future research should implement manipulation checks to confirm that recalled scenarios match the expected patterns during the recall. Subjects can be asked to write down their recalled scenarios in advance and to confirm what emotion they feel during the recall.

Across our studies, we also chose to randomize the presentation of stimuli in order to avoid trial effects biasing the findings of our studies. Some emotion literature suggests that part of perceiving an emotion involves feeling that emotion through a process called mirroring and that perceived emotions can influence one’s own mood through emotion contagion (Barsade et al. 2003). Thus, it is possible that presentation of emotion trials differing on valence, for example, could bias future trials. For example, if one were to receive several negative emotion stimuli in a row, perhaps it would decrease the accuracy of the next subsequent positive stimuli presented. This could have real-world implications as an individual moves across situations where the valence of emotions present is very different (e.g., leaving a hostile meeting, you may be more prone to mis-perceive negative emotions in others). Future research should examine independence/non-independence of trials when presenting emotion stimuli. 

Finally, our study found inconsistent correlations between the MET and age. In study 3, the MET had a significant negative correlation with age (*r* = −0.47, *p* < .05) such that older participants scored lower on the MET. This finding was driven by a bimodal sample that consisted of younger students and some older non-student adults. Findings in Study 4, however, showed that the MET was correlated positively with age (*r* = 0.15, *p* < .05). Prior research has shown curvilinear relationships between the MSCEIT and age (Cabello et al. 2016), and future research should examine the relationship between the MET and age. 

### 7.2. Practical Contributions and Research Implications

A key goal of this research was to develop and validate a novel measure of naturalistic emotion perception for diverse individuals that could be used for research purposes in the U.S. As a part of this goal, the MET will be made freely available online to researchers interested in using it. Although many assessments of emotion perception exist, the lack of valid and free assessments has limited the progress of emotion ability research. A core practical contribution is thus to make the MET freely available and accessible via the web for research purposes. 

Another practical contribution is that the MET holds the potential to substantially improve the quality of information measured about one’s emotion perception ability. It helps to address the serious threat to validity of in-group biases in the stimuli to improve fairness in the assessment. Additionally, as we found in Figure 1, the MET outperforms other tests of emotion perception, as it provides more information about emotion perception ability and includes less measurement error. Relatedly, the MET consistently gives information about emotion perception ability across a wide range of latent ability. This practical contribution means that the MET can provide researchers with valuable information at low, moderate, and high perception ability levels. For example, the MET is appropriate to use in populations with putatively high emotion perception requirements (e.g., customer service, education, nursing) and low emotion perception requirements (e.g., mechanics, engineering, software development). 

## 8. Conclusions

Our research theorized and found evidence in support of a meso-expression paradigm for measuring emotion perception. We show that meso-expression—the moderate-intensity, conscious emotion expressions of everyday life—can be reliably perceived. We developed the MET, a psychometrically rigorous and race- and gender-balanced measure of meso-expression. Across four studies, we demonstrate the validity of the MET and make it freely available for research.

## Figures and Tables

**Table 1:** Confusion Matrix for Nonverbal Stimuli.

	Amu	Ang	Anx	Awe	Bor	Cmp	Con	Dis	Emb	Fea	Joy	Neu	Pri	Rel	Sad	Sha	Sup	Sym
Amusement (Amu)	0.92	0.03	0.12	0.26	0.06	0.27	0.63	0.07	0.30	0.05	0.88	0.33	0.39	0.39	0.02	0.09	0.21	0.09
Anger (Ang)	0.04	0.93	0.31	0.07	0.33	0.74	0.21	0.69	0.13	0.41	0.04	0.59	0.09	0.15	0.49	0.36	0.04	0.11
Anxiety (Anx)	0.03	0.42	0.85	0.12	0.38	0.41	0.16	0.50	0.59	0.74	0.06	0.60	0.08	0.25	0.66	0.65	0.23	0.18
Awe	0.49	0.06	0.51	0.76	0.24	0.35	0.43	0.26	0.23	0.51	0.49	0.75	0.24	0.60	0.23	0.18	0.80	0.20
Boredom (Bor)	0.03	0.34	0.39	0.10	0.90	0.62	0.17	0.60	0.39	0.20	0.05	0.65	0.07	0.35	0.65	0.59	0.13	0.21
Contempt (Cmp)	0.05	0.85	0.35	0.09	0.47	0.77	0.28	0.80	0.25	0.33	0.04	0.65	0.14	0.15	0.47	0.48	0.10	0.16
Content (Con)	0.57	0.05	0.23	0.27	0.59	0.50	0.82	0.24	0.18	0.06	0.66	0.80	0.41	0.69	0.21	0.14	0.08	0.30
Disgust (Dis)	0.03	0.67	0.46	0.18	0.31	0.70	0.18	0.91	0.27	0.47	0.03	0.58	0.08	0.10	0.56	0.41	0.24	0.14
Embarrassment (Emb)	0.70	0.09	0.62	0.27	0.27	0.38	0.43	0.40	0.80	0.41	0.49	0.48	0.29	0.49	0.41	0.71	0.32	0.32
Fear (Fea)	0.03	0.48	0.78	0.26	0.19	0.35	0.10	0.49	0.31	0.89	0.05	0.58	0.04	0.20	0.57	0.31	0.65	0.17
Joy	0.82	0.02	0.22	0.34	0.09	0.29	0.74	0.06	0.22	0.09	0.89	0.55	0.49	0.54	0.03	0.03	0.19	0.11
Neutral (Neu)	0.01	0.46	0.26	0.14	0.67	0.54	0.35	0.32	0.04	0.26	0.06	0.96	0.12	0.13	0.35	0.14	0.18	0.12
Pride (Pri)	0.72	0.11	0.35	0.31	0.17	0.42	0.78	0.12	0.24	0.19	0.69	0.66	0.78	0.68	0.05	0.07	0.20	0.18
Relief (Rel)	0.11	0.09	0.51	0.16	0.72	0.43	0.38	0.47	0.22	0.30	0.14	0.56	0.10	0.92	0.50	0.34	0.14	0.31
Sadness (Sad)	0.03	0.19	0.49	0.12	0.43	0.39	0.17	0.45	0.33	0.54	0.08	0.71	0.04	0.15	0.92	0.63	0.11	0.42
Shame (Sha)	0.03	0.30	0.49	0.04	0.32	0.40	0.12	0.54	0.68	0.46	0.07	0.47	0.03	0.08	0.84	0.87	0.11	0.30
Surprise (Sup)	0.45	0.02	0.39	0.71	0.22	0.24	0.37	0.21	0.23	0.48	0.49	0.51	0.10	0.70	0.13	0.09	0.91	0.13
Sympathy (Sym)	0.04	0.16	0.53	0.15	0.55	0.47	0.30	0.40	0.38	0.50	0.05	0.70	0.04	0.34	0.83	0.62	0.13	0.77

Notes: The table above contains the *pi* values for each intended emotion across all stimuli. The rows show the intended emotion while each column shows the *pi* value for each of the possible emotion labels. The diagonal of the matrix (shaded in grey) shows the hit rate for each intended emotion category, while values off-diagonal represent confusions (i.e., false positives). *pi* values on the diagonal above 0.50 are considered to be recognized above chance. On average, each intended emotion category was recognized above chance across the nonverbal stimuli.

**Table 2:** Confusion Matrix for Verbal Stimuli.

	Amu	Ang	Anx	Awe	Bor	Cmp	Con	Dis	Emb	Fea	Joy	Neu	Pri	Rel	Sad	Sha	Sup	Sym
Amusement (Amu)	0.78	0.08	0.33	0.26	0.36	0.30	0.56	0.17	0.28	0.27	0.82	0.80	0.32	0.41	0.49	0.13	0.49	0.19
Anger (Ang)	0.20	0.59	0.38	0.13	0.66	0.63	0.53	0.45	0.22	0.27	0.33	0.89	0.40	0.31	0.50	0.25	0.13	0.40
Anxiety (Anx)	0.17	0.09	0.79	0.19	0.49	0.39	0.50	0.25	0.45	0.66	0.35	0.83	0.20	0.45	0.49	0.34	0.44	0.39
Awe	0.19	0.03	0.61	0.49	0.39	0.27	0.56	0.17	0.27	0.53	0.57	0.83	0.29	0.57	0.38	0.28	0.66	0.58
Boredom (Bor)	0.17	0.21	0.45	0.19	0.87	0.52	0.43	0.41	0.25	0.29	0.19	0.84	0.24	0.36	0.61	0.33	0.17	0.21
Contempt (Cmp)	0.18	0.27	0.42	0.22	0.73	0.56	0.59	0.43	0.20	0.29	0.34	0.88	0.26	0.35	0.57	0.28	0.34	0.42
Content (Con)	0.29	0.08	0.39	0.31	0.62	0.40	0.67	0.16	0.26	0.28	0.50	0.90	0.27	0.43	0.58	0.25	0.21	0.50
Disgust (Dis)	0.16	0.26	0.48	0.15	0.69	0.59	0.50	0.46	0.30	0.35	0.31	0.89	0.25	0.33	0.60	0.28	0.25	0.34
Embarrassment (Emb)	0.41	0.09	0.65	0.26	0.64	0.41	0.53	0.26	0.48	0.57	0.52	0.83	0.21	0.41	0.56	0.34	0.30	0.43
Fear (Fea)	0.18	0.09	0.77	0.25	0.43	0.22	0.44	0.15	0.37	0.77	0.31	0.81	0.19	0.48	0.62	0.33	0.38	0.43
Joy	0.67	0.06	0.40	0.23	0.39	0.31	0.63	0.13	0.19	0.21	0.80	0.85	0.40	0.39	0.35	0.17	0.45	0.39
Neutral (Neu)	0.23	0.18	0.18	0.19	0.58	0.42	0.68	0.26	0.13	0.08	0.48	0.94	0.39	0.34	0.40	0.14	0.18	0.36
Pride (Pri)	0.35	0.20	0.27	0.20	0.57	0.47	0.66	0.29	0.11	0.16	0.66	0.90	0.51	0.29	0.51	0.21	0.35	0.30
Relief (Rel)	0.31	0.15	0.59	0.29	0.69	0.47	0.50	0.38	0.31	0.45	0.51	0.75	0.26	0.73	0.54	0.30	0.31	0.47
Sadness (Sad)	0.07	0.09	0.63	0.14	0.60	0.35	0.35	0.21	0.34	0.70	0.18	0.82	0.12	0.30	0.81	0.48	0.20	0.51
Shame (Sha)	0.12	0.07	0.58	0.19	0.71	0.37	0.46	0.24	0.40	0.57	0.21	0.85	0.14	0.36	0.72	0.46	0.23	0.48
Surprise (Sup)	0.58	0.05	0.46	0.37	0.38	0.22	0.58	0.12	0.22	0.34	0.80	0.77	0.36	0.51	0.23	0.12	0.76	0.34
Sympathy (Sym)	0.17	0.06	0.45	0.25	0.53	0.37	0.59	0.19	0.29	0.53	0.42	0.86	0.18	0.45	0.64	0.33	0.36	0.70

Notes: The table above contains the *pi* values for each intended emotion across all stimuli. The rows show the intended emotion while each column shows the *pi* value for each of the possible emotion labels. The diagonal of the matrix (shaded in grey) shows the hit rate for each intended emotion category, while values off-diagonal represent confusions (i.e., false positives). *pi* values on the diagonal above 0.50 are considered to be recognized above chance. On average, each intended emotion category was recognized above chance across the stimuli.

**Table 3:** Confusion Matrix for Concealed Stimuli.

	Amu	Ang	Anx	Awe	Bor	Cmp	Con	Dis	Emb	Fea	Joy	Neu	Pri	Rel	Sad	Sha	Sup	Sym
Concealed emotion																		
Anger (Ang)	0.61	0.33	0.22	0.17	0.45	0.40	0.75	0.34	0.15	0.15	0.85	0.80	0.20	0.55	0.26	0.12	0.19	0.24
Content (Con)	0.38	0.81	0.29	0.16	0.50	0.62	0.50	0.58	0.19	0.21	0.56	0.82	0.20	0.37	0.33	0.22	0.37	0.26
Sadness (Sad)	0.71	0.10	0.15	0.18	0.22	0.22	0.71	0.13	0.17	0.11	0.92	0.65	0.15	0.49	0.40	0.15	0.17	0.29
Joy	0.44	0.26	0.36	0.15	0.47	0.37	0.61	0.31	0.33	0.34	0.70	0.85	0.17	0.38	0.72	0.34	0.27	0.39
Displayed emotion																		
Content (Con)	0.48	0.76	0.64	0.10	0.58	0.57	0.20	0.67	0.39	0.58	0.50	0.35	0.11	0.26	0.73	0.34	0.28	0.09
Anger (Ang)	0.62	0.82	0.51	0.14	0.48	0.58	0.27	0.66	0.32	0.56	0.63	0.40	0.11	0.26	0.52	0.29	0.43	0.07
Joy	0.50	0.32	0.64	0.09	0.55	0.35	0.24	0.41	0.49	0.55	0.56	0.36	0.11	0.28	0.89	0.47	0.24	0.14
Sadness (Sad)	0.67	0.33	0.56	0.19	0.38	0.27	0.30	0.44	0.47	0.66	0.70	0.43	0.15	0.23	0.83	0.47	0.35	0.17
	
Type of Concealed Expression						*Mean pi*												
Felt anger but displayed contentment						0.23												
Felt contentment but displayed anger						0.85												
Felt sadness but displayed joy						0.83												
Felt joy but displayed sadness						0.95												

Notes: The table above contains the *pi* values for each intended concealed and displayed emotion across all concealed stimuli. The rows show the intended concealed or displayed emotion while each column shows the *pi* value for each of the possible emotion labels. The values shaded in grey show the hit rate for each intended emotion category. Below the confusion matrix, we present the average *pi* values for selecting both the correct concealed and displayed emotion. Values above 0.50 are considered to be recognized above chance.

**Table 4:** Statistics for Nonverbal Items.

Item Name	Emotion	Item Information	Factor Loadings	*χ* ^2^	*d.f.*	*p*	*χ*^2^/*d.f.*
NV1	Amusement	1.26	0.54	160.27	141	0.13	1.14
NV2	Amusement	0.94	0.48	154.68	140	0.19	1.1
NV3	Amusement	0.95	0.48	162.45	143	0.13	1.14
NV4	Amusement	0.73	0.38	156.58	158	0.52	0.99
NV5	Amusement	0.93	0.44	153.74	157	0.56	0.98
NV6	Amusement	0.99	0.46	173.61	146	0.06	1.19
NV7	Boredom	0.72	0.42	171.35	148	0.09	1.16
NV8	Boredom	0.72	0.41	169.86	155	0.2	1.1
NV9	Boredom	0.49	0.32	169.5	163	0.35	1.04
NV10	Content	0.59	0.35	132.62	158	0.93	0.84
NV11	Content	0.65	0.37	146.13	153	0.64	0.96
NV12	Content	0.65	0.4	194.65	140	0	1.39
NV13	Content	0.83	0.42	211.77	152	0	1.39
NV14	Disgust	0.75	0.45	160.62	141	0.12	1.14
NV15	Disgust	0.77	0.41	144.67	155	0.71	0.93
NV16	Embarrassment	0.61	0.38	137.34	155	0.84	0.89
NV17	Embarrassment	0.92	0.44	151.92	152	0.49	1
NV18	Embarrassment	0.68	0.38	170.56	152	0.14	1.12
NV19	Relief	0.51	0.38	166.48	143	0.09	1.16
NV20	Relief	0.65	0.46	147.98	127	0.1	1.17
NV21	Sad	0.65	0.41	131.95	139	0.65	0.95
NV22	Sad	0.46	0.32	175.37	152	0.09	1.15
NV23	Shame	0.74	0.39	181.41	157	0.09	1.16
NV24	Shame	0.6	0.37	160.47	149	0.25	1.08
NV25	Shame	0.68	0.39	153.55	154	0.5	1
NV26	Surprise	0.37	0.29	161.45	158	0.41	1.02
NV27	Surprise	0.74	0.41	128.21	153	0.93	0.84
NV28	Sympathy	1.05	0.5	197.73	144	0	1.37
NV29	Sympathy	0.77	0.41	143.07	149	0.62	0.96
NV30	Sympathy	0.9	0.47	126.54	142	0.82	0.89
NV31	Sympathy	0.84	0.41	145.04	155	0.71	0.94

**Table 5:** Statistics for Verbal Items.

Item Name	Emotion	Item Information	Factor Loadings	*χ* ^2^	*d.f.*	*p*	*χ*^2^/*d.f.*
V2	Amusement	0.49	0.31	73.37	53	0.03	1.38
V3	Amusement	0.32	0.27	77.53	52	0.01	1.49
V5	Anger	0.87	0.45	61.88	51	0.14	1.21
V6	Anger	1.10	0.47	78.89	52	0.01	1.52
V9	Boredom	0.89	0.45	56.5	50	0.24	1.13
V10	Boredom	0.65	0.37	46.21	53	0.73	0.87
V13	Content	0.35	0.27	48.11	54	0.70	0.89
V14	Content	0.46	0.33	75.84	49	0.01	1.55
V17	Disgust	0.93	0.46	66.34	51	0.07	1.30
V19	Disgust	1.27	0.51	63.87	48	0.06	1.33
V22	Embarrassment	0.49	0.31	59.12	54	0.29	1.09
V23	Embarrassment	0.55	0.33	52.56	53	0.49	0.99
V25	Fear	0.80	0.39	54.98	52	0.36	1.06
V28	Fear	0.42	0.29	60.83	55	0.27	1.11
V33	Sadness	0.63	0.36	74.03	53	0.03	1.40
V34	Sadness	0.25	0.22	61.67	55	0.25	1.12
V38	Surprise	0.83	0.42	60.87	52	0.19	1.17
V39	Surprise	0.78	0.4	94.03	53	0.00	1.77
V41	Sympathy	0.97	0.44	60.5	52	0.20	1.16
V42	Sympathy	0.57	0.35	53.94	54	0.48	1.00

**Table 6:** Concealed Item Statistics.

Item Name	Emotion	Item Information	Factor Loadings	*χ* ^2^	*d.f.*	*p*	*χ*^2^/*d.f.*
C1	Anger X Content	1.66	0.54	77.15	67	0.19	1.15
C2	Anger X Content	0.48	0.32	79.24	64	0.09	1.24
C3	Anger X Content	0.95	0.42	75.04	61	0.11	1.23
C4	Anger X Content	0.98	0.44	85.39	60	0.02	1.42
C5	Anger X Content	0.96	0.42	79.68	67	0.14	1.19
C6	Content X Anger	0.77	0.39	153.81	76	0.00	2.02
C7	Joy X Sadness	0.83	0.40	158.21	79	0.00	2.00
C8	Joy X Sadness	0.52	0.32	155.54	78	0.00	1.99
C9	Sad X Joy	1.75	0.57	79.24	62	0.07	1.28
C10	Sad X Joy	1.58	0.54	75.31	57	0.05	1.32
C11	Sad X Joy	1.10	0.46	63.79	67	0.59	0.95
C12	Sad X Joy	1.52	0.53	92.94	72	0.05	1.29
C13	Sad X Joy	1.22	0.48	72.75	64	0.21	1.14

**Table 7:** Differential Item Functioning.

	*d-dif*
Non-Verbal	
Asian versus Hispanic	0.10
Black versus Hispanic	0.11
Black versus Asian	0.11
White versus Black	0.20
White versus Hispanic	0.13
White versus Asian	0.14
Men versus Women	0.12
Verbal	
Asian versus Hispanic	0.29
Black versus Hispanic	0.14
Black versus Asian	0.34
White versus Black	0.11
White versus Hispanic	0.23
White versus Asian	0.40
Men versus Women	0.12
Concealed	
Asian versus Hispanic	0.15
Black versus Hispanic	0.16
Black versus Asian	0.17
White versus Black	0.23
White versus Hispanic	0.12
White versus Asian	0.19
Men versus Women	0.08

**Table 8:** Convergent and Divergent Validity.

	1	2	3	4	5	6	7	8	9
1. MET total score	1								
2. MET Non-Verbal	0.85 *	1							
3. MET Concealed	0.82 *	0.48 *	1						
4. MET Verbal	0.78 *	0.51 *	0.50 *	1					
5. GERT	0.68 *	0.59 *	0.53 *	0.52 *	1				
6. DANVA total score	0.59 *	0.50 *	0.41 *	0.55 *	0.62 *	1			
7. DANVA faces	0.39 *	0.34 *	0.26 *	0.34 *	0.41 *	0.73 *	1		
8. DANVA voices	0.55 *	0.45 *	0.40 *	0.52 *	0.56 *	0.86 *	0.29 *	1	
9. WPTQ	0.36 *	0.34 *	0.27 *	0.29 *	0.46 *	0.25 *	0.17 *	0.23 *	1
10. Age	−0.47 *	−0.35 *	−0.32 *	−0.50 *	−0.45 *	−0.29 *	−0.13	−0.32 *	−0.32 *

Notes: * *p* < .05.

**Table 9:** MET Correlates.

	1	2	3	4	5	6	7	8	9	10	11	12	13	14	15
1. MET total score	1														
2. MET Non-Verbal	0.92 *	1													
3. MET Concealed	0.82 *	0.57 *	1												
4. MET Verbal	0.83 *	0.67 *	0.49 *	1											
5. Social well-being	0.16 *	0.19 *	0.06	0.12 *	1										
6. Empathic con.	0.38 *	0.38 *	0.24 *	0.31 *	0.46 *	1									
7. Social connect.	0.28 *	0.29 *	0.17 *	0.23 *	0.59 *	0.36 *	1								
8. Alexithymia	−0.42 *	−0.42 *	−0.24 *	−0.37 *	−0.49 *	−0.41 *	−0.75 *	1							
9. Stress	−0.13 *	−0.17 *	−0.05	−0.09 *	−0.42 *	−0.13 *	−0.55 *	0.56 *	1						
10. Depression	−0.33 *	−0.35 *	−0.19 *	−0.27 *	−0.41 *	−0.27 *	−0.70 *	0.68 *	0.65 *	1					
11. Anxiety	−0.33 *	−0.34 *	−0.20 *	−0.27 *	−0.37 *	−0.23 *	−0.67 *	0.65 *	0.66 *	0.87 *	1				
12. Positive rel.	−0.10 *	−0.09 *	−0.07 *	−0.10 *	0.36 *	0.19 *	0.01	0.07	−0.01	0.10 *	13 *	1			
13. Negative rel.	−0.54 *	−0.53 *	−0.34 *	−0.48 *	−0.23 *	−0.35 *	−0.53 *	0.61 *	0.35 *	0.57 *	0.55 *	0.26 *	1		
14. Age	0.15 *	0.24 *	−0.03	0.11 *	0.17 *	0.09 *	0.17 *	0.21 *	−0.29 *	−0.29 *	0−.28 *	−0.02	−0.29 *	1	
15. Gender	−0.30 *	−0.26 *	−0.17 *	−0.26 *	−0.07 *	−0.18 *	−0.11 *	0.20 *	−0.02	0.10 *	0.10 *	0.08 *	0.26 *	0.09 *	1

Notes: * *p* < .05.

**Table 10:** MET × Gender Interactions.

	*β*	*R* ^2^
Social Well-being		
MET score	0.35 *	
Gender	−0.14 *	
Gender × MET score	−0.26 ^†^	0.03
Empathic concern		
MET score	0.59 *	
Gender	−0.20 *	
Gender × MET score	−0.30 *	0.16
Social connectedness		
MET score	0.20 ^†^	
Gender	0.01	
Gender × MET score	0.10	0.08
Alexithymia		
MET score	−0.30 *	
Gender	0.05	
Gender × MET score	−0.12	0.18
Stress		
MET score	0.24 *	
Gender	−0.26 *	
Gender × MET score	−0.51 *	0.04
Depression		
MET score	0.02	
Gender	−0.17 *	
Gender × MET score	−0.44 *	0.12
Anxiety		
MET score	−0.03	
Gender	−0.14 *	
Gender × MET score	−0.38 *	0.12
Positive relationships		
MET score	0.12	
Gender	−0.08	
Gender × MET score	−0.34 *	0.02
Negative relationships		
MET score	−0.31 *	
Gender	0.01	
Gender × MET score	−0.26 *	0.31

Notes: * *p* < .05, ^†^ *p* < .10.

**Table 11:** MET Correlates for Women and Men.

	*r*
Women MET scores with	
Social well-being	0.21 *
Empathic concern	0.40 *
Social connectedness	0.25 *
Alexithymia	−0.35 *
Stress	0.00
Depression	−0.20 *
Anxiety	−0.21 *
Neg Social interactions	−0.46 *
Pos Social interactions	0.02
Men MET scores with	
Social well-being	0.06
Empathic concern	0.28 *
Social connectedness	0.27 *
Alexithymia	−0.41 *
Depression	−0.43 *
Stress	−0.33 *
Anxiety	−0.41 *
Neg Social interactions	−0.54 *
Pos Social interactions	−0.19 *

Notes: * *p* < .05.

## Data Availability

Data is available at https://osf.io/7czyp/?view_only=ba134383e47d4bdbb5da2da40298d5a5 (accessed on 1 July 2023).

## Notes

1 The most popular assessment of EI is the Mayer-Salovey-Caruso Emotional Intelligence Test (MSCEIT; Mayer 2002) which has over 3000 citations, and evidence of validity across cultures. Assessments of emotion perception include the Japanese and Caucasian Facial Expressions of Emotion instrument (JACFEE; Matsumoto and Ekman 1988), the Diagnostic Analysis of Nonverbal Accuracy (DANVA; Nowicki and Duke 1994) and, more recently, the Geneva Emotion Recognition Test (GERT; Schlegel and Scherer 2016). These assessments all examine macro-expressions.

2 In this study, we were interested in both male and female expressors and we included actors who identified as male or female. We also included actors who identified as Asian, Black, Hispanic, and White. We note that other gender and racial identities exist and that the groups included in our study were a starting point to better understand the interplay of intersectionality in emotion perception ability.

3 Each participant rated 4 randomly selected nonverbal Caucasian stimuli, 4 randomly selected nonverbal African-American stimuli, 4 randomly selected nonverbal Asian stimuli, 4 randomly selected nonverbal Hispanic stimuli, 3 randomly selected verbal Caucasian stimuli, 3 randomly selected verbal African-American stimuli, 3 randomly selected verbal Hispanic stimuli, 3 randomly selected verbal Asian stimuli, 1 randomly selected concealed Caucasian stimuli, 1 randomly selected concealed African-American stimuli, 1 randomly selected concealed Hispanic stimuli, and 1 randomly selected concealed Asian stimuli.

4 Some have argued that perceivers may simply select the same emotion label repeatedly, which can lead to artificially high recognition rates above chance (Laukka et al. 2016; Wagner 1993; See also Gendron et al. 2018). For example, if perceivers always select ‘anger,’ all anger stimuli will be recognized above chance. Research shows, however, that perceivers are actually failing to distinguish anger from other emotions (Elfenbein and Ambady 2003). In this study we were interested in patterns of confusions between emotions which may share phenomenological and cognitive appraisal similarities. In fact, we intentionally included emotions which may be easily confused (e.g., embarrassment and shame) during our assessment development with the intention of both: (1) increasing the difficulty of the test we created, and (2) giving perceivers credit for answers that are close to the intended emotion (i.e., embarrassment may be a plausible answer for a shame stimuli). This Graded Response Model approach, which we elaborate more on in Study 2, fundamentally treats responses as having a continuum of correctness or accuracy (as opposed to a correct/incorrect dichotomy). We believe that since different discrete emotions share phenomenological and appraisal similarities, a graded approach is more appropriate for our study rather than controlling for confusions or false positives. In other words, we treat confusions for emotions that are similar as evidence of emotion perception ability and as evidence that emotions concepts are a fuzzy set.

5 Because the expressors are concealing their true emotion, it was expected that perceivers would view the displays as less authentic in our believability rating.

6 These results are for the nonverbal and verbal stimuli. The concealed displays were also rated as moderate intensity (2.73 out of 5) but as expected, rated low on believability rating (1.05 out of 5) because these displays involve concealment of emotions.

7 We calculated the total amount of information provided by each item by calculating the items’ information function from *θ* = −3 to *θ* = +3. This area under the item information curve allows us to compare the relative value of each item in the test to determine which items contribute the most information, and therefore, should be retained.

8 We use the mean item information as a starting place to examine whether an item relatively improves the information value of our assessment rather than as a rule-of-thumb cut-off. See below.

9 Because the Chi-square test is sensitive to sample size and may be insensitive to model misfit, it is recommended to examine the ratio between chi-square and degrees of freedom (Tay et al. 2011).

## Supplementary Materials

The following supporting information can be downloaded at: https://www.mdpi.com/article/10.3390/jintelligence11070145/s1, Table S1: Study 1 Item-level Confusion Matrix and Selected Distractors; Table S2: Study 2 Item-level Scoring and Confusions.
