# Supplementary material for: The Meso-Expression Test (MET): A Novel Assessment of Emotion Perception
Source: J Intell. 2023 Jul 19;11(7):145. doi: 10.3390/jintelligence11070145 (PMC10381771; doi:10.3390/jintelligence11070145)
Supplement: Supplementary file 1 [file jintelligence-11-00145-s001.zip › jintelligence-2320361-supplementary.pdf]

Supplemental Material

Item-level confusion and distractor methodology and results

We selected distractors at the item level based on the confusions of related emotion labels in Study 1 which we present in Table S1. That is, distractors selection emphasized the specific expression in the stimuli, and therefore distractors between items were varied. We also, emphasized selecting distractors that were ‘plausible’ in that they were confused with the target emotion.

To construct items, we paired each stimulus in table S1 with the correct answer, at least one partially correct answer that was frequently confused with the target emotion and may have shared characteristics with the target emotion. Finally, we chose plausible incorrect distractors which were confused at least some of the time with the target emotion and may share some features with that emotion. We note that we used this procedure as a heuristic to guide our decision making on identifying distractors and choosing which answers should receive credit.

For example, item 1 in the non-verbal MET is based on an amusement stimulus (Table S1). As can be seen in the table, this stimulus was recognized above chance as amusement (*pi*=.96). However, it was also frequently confused with Joy (*pi*= .87). Notably, Joy shares similar valence and arousal with amusement and facial features such as corrugator muscle activation (i.e., smiling; Cordaro et al., 2020). Additionally, the labels content (*pi*=.38), relief (*pi*=.33), pride (*pi*=.27) and surprise (*pi*=.27) were also frequent confusions (but less so than Joy) and did not necessarily share the same arousal of amusement (content and relief are low arousal and surprise is very high arousal and ambiguous valence). Consistent with the findings above, we considered Amusement to be the correct answer, Joy to be a partially correct distractor, and content, relief, pride, and surprise to be plausible incorrect distractors (i.e., they were selected at least some of the time) for which we did not give credit. This procedure was repeated for each item.

For the nonverbal and verbal MET sub-tests, we coded polytomous responses in the Graded Response Model (GRM) as (2) Correct answer, (1) partially correct distractor, or (0) incorrect distractor. For the concealed MET sub-test, we coded items polytomous in the GRM as (3) correct answer [i.e., correctly labeling the concealed and expressed emotions], (2) partially correct answer but labels were flipped [i.e., labeled the concealed emotion as the expressed emotion and vice versa], (1) partially correct answer but only identifying one emotion label correctly [i.e., either the labeled or expressed emotion was correct, and the second label was an incorrect wildcard distractor], (0) incorrect distractors selected for both concealed and expressed emotions. All item codings are listed in table S-2.

In Table S2, we show the item level results from Study 2. Across items we replicate results from study 1 and find that correct emotion labels were all selected above chance. Additionally, we find that partially correct distractors were selected frequently and, in most cases, more so than the plausible incorrect distractors as expected.

Table S1 Study 1 Item-level Confusion Matrix and Selected Distractors

Item #	Type	Amu	Ang	Anx	Awe	Bor	Cmp	Con	Dis	Emb	Fea	Joy	Neu	Pri	Rel	Sad	Sha	Sup	Sym	Selected Distractors	Partially correct distractor	Incorrect distractor
1	NV AMU	0.96	0.00	0.00	0.19	0.00	0.11	0.38	0.00	0.00	0.00	0.87	0.11	0.27	0.33	0.00	0.00	0.27	0.11	Joy, Content, Relief, Pride, Surprise	Joy	Content, Relief, Pride, Surprise
2	NV AMU	0.97	0.00	0.23	0.13	0.00	0.37	0.37	0.00	0.31	0.00	0.73	0.31	0.43	0.13	0.00	0.23	0.31	0.00	Joy, Pride, Content, Contempt, Surprise	Joy	Content, Contempt, Surprise
3	NV AMU	0.95	0.00	0.14	0.32	0.00	0.45	0.50	0.39	0.24	0.00	0.71	0.39	0.32	0.50	0.14	0.14	0.24	0.00	Joy, Content, Relief, Contempt, Awe	Joy	Content, Relief, Contempt, Awe
4	NV AMU	0.96	0.00	0.11	0.00	0.00	0.11	0.28	0.00	0.20	0.00	0.86	0.28	0.11	0.48	0.11	0.00	0.34	0.11	Joy, Relief, Content, Surprise, Embarrassment	Joy	Relief, Content, Surprise, Embarrassment
5	NV AMU	0.97	0.00	0.00	0.12	0.00	0.12	0.45	0.12	0.21	0.00	0.82	0.28	0.21	0.28	0.00	0.00	0.00	0.00	Joy, Content, Relief, Pride, Embarrassment	Joy	Content, Relief, Pride, Embarrassment
6	NV AMU	0.96	0.00	0.00	0.00	0.00	0.13	0.37	0.00	0.13	0.13	0.88	0.22	0.13	0.43	0.00	0.00	0.30	0.00	Joy, Relief, Content, Surprise, Fear	Joy	Relief, Content, Surprise, Fear
7	NV BOR	0.00	0.14	0.25	0.00	0.94	0.25	0.14	0.51	0.00	0.00	0.00	0.88	0.00	0.00	0.55	0.33	0.14	0.14	Sadness, Disgust, Shame, Anxiety, Contempt	Sadness, Disgust	Shame, Anxiety, Contempt
8	NV BOR	0.28	0.21	0.45	0.11	0.95	0.65	0.40	0.45	0.55	0.00	0.11	0.52	0.21	0.11	0.00	0.28	0.00	0.11	Contempt, Embarrassment, Disgust, Anxiety, Content	Contempt, Embarrassment	Disgust, Anxiety, Content
9	NV BOR	0.00	0.12	0.60	0.12	0.88	0.47	0.12	0.30	0.12	0.22	0.12	0.55	0.12	0.30	0.88	0.55	0.00	0.22	Anxiety, Shame, Contempt, Disgust, relief	Anxiety, Shame	Contempt, Disgust, relief
10	NV CON	0.66	0.00	0.14	0.33	0.33	0.55	0.86	0.00	0.00	0.00	0.76	0.84	0.40	0.33	0.00	0.00	0.14	0.25	Joy, Amusement, Contempt, Pride, Boredom	Joy, Amusement	Contempt, Pride, Boredom
11	NV CON	0.50	0.00	0.12	0.46	0.36	0.50	0.92	0.12	0.00	0.00	0.60	0.65	0.22	0.78	0.22	0.12	0.00	0.00	Relief, Joy, Amusement, Contempt, Awe	Relief, Joy, Amusement	Contempt, Awe
12	NV CON	0.13	0.00	0.31	0.13	0.23	0.53	0.83	0.13	0.23	0.13	0.62	0.93	0.31	0.13	0.31	0.13	0.00	0.23	Joy, Contempt, Anxiety, Sadness, Amusement	Joy	Contempt, Anxiety, Sadness, Amusement
13	NV CON	0.64	0.00	0.00	0.41	0.00	0.41	0.90	0.00	0.00	0.12	0.75	0.80	0.50	0.53	0.00	0.00	0.21	0.12	Joy, Amusement, Relief, Awe, Contempt	Joy, Amusement, Relief	Awe, Contempt
14	NV DIS	0.14	0.46	0.00	0.14	0.14	0.74	0.00	0.97	0.00	0.14	0.00	0.25	0.14	0.00	0.33	0.25	0.14	0.00	Contempt, Anger, Sadness, Shame, Fear	Contempt	Anger, Sadness, Shame, Fear
15	NV DIS	0.00	0.29	0.12	0.21	0.00	0.46	0.12	0.96	0.12	0.50	0.00	0.00	0.00	0.12	0.77	0.50	0.29	0.12	Sadness, Shame, Fear, Contempt, Anger	Sadness	Shame, Fear, Contempt, Anger
16	NV EMB	0.71	0.13	0.38	0.00	0.00	0.23	0.23	0.23	0.79	0.38	0.32	0.38	0.00	0.32	0.67	0.90	0.00	0.38	Amusement, Sadness, Anxiety, Sympathy, Fear	Amusement, Sadness	Anxiety, Sympathy, Fear
17	NV EMB	0.77	0.00	0.87	0.23	0.00	0.00	0.13	0.13	0.87	0.52	0.31	0.23	0.00	0.23	0.00	0.58	0.37	0.13	Amusement, Shame, Fear, Surprise, Joy	Amusement, Shame, Fear	Surprise, Joy
18	NV EMB	0.70	0.00	0.48	0.23	0.00	0.13	0.23	0.38	0.93	0.13	0.23	0.23	0.13	0.31	0.59	0.72	0.13	0.00	Shame, Amusement, Sadness, Anxiety, Disgust	Shame, Amusement	Sadness, Anxiety, Disgust
19	NV REL	0	0	0.26	0.34	0.76	0.26	0.15	0.26	0.15	0	0.15	0.15	0	0.97	0.15	0	0.26	0.41	Boredom, Sympathy, Awe, Anxiety, Contempt	Boredom	Sympathy, Awe, Anxiety, Contempt
20	NV REL	0	0	0.66	0	0.46	0.12	0.12	0.22	0	0.12	0	0.36	0	0.98	0.22	0	0	0.12	Anxiety, Boredom, Sadness, Disgust, Fear	Anxiety	Boredom, Sadness, Disgust, Fear
21	NV SAD	0	0.11	0.28	0.11	0	0.11	0	0.11	0.21	0.4	0	0.61	0	0.21	0.97	0.52	0	0.49	Shame, Sympathy, Fear, Anxiety, Embarrassment	Shame	Sympathy, Fear, Anxiety, Embarrassment
22	NV SAD	0	0	0.54	0	0.13	0.24	0	0.63	0.39	0.54	0	0.39	0	0.24	0.95	0.5	0	0.65	Sympathy, Disgust, Fear, Anxiety, Shame	Sympathy	Disgust, Fear, Anxiety, Shame

Item #	Type	Amu	Ang	Anx	Awe	Bor	Cmp	Con	Dis	Emb	Fea	Joy	Neu	Pri	Rel	Sad	Sha	Sup	Sym	Selected Distractors	Partially correct distractor	Incorrect distractor
23	NV SHA	0.14	0.14	0.25	0	0.14	0.4	0	0.33	0.83	0.33	0.14	0.25	0	0	0.76	0.93	0	0.14	Embarrassment, Sadness, Contempt, Disgust, Fear	Embarrassment	Sadness, Contempt, Disgust, Fear
24	NV SHA	0.14	0.14	0.33	0	0.5	0.45	0	0.24	0.5	0	0	0.58	0	0.14	0.93	0.81	0.14	0.45	Embarrassment, Boredom, Sympathy, Contempt, Anxiety	Embarrassment	Boredom, Sympathy, Contempt, Anxiety
25	NV SHA	0	0	0.11	0	0	0.11	0.11	0.11	0.68	0.28	0	0.11	0.11	0.11	0.92	0.92	0.11	0.2	Embarrassment, Fear, Sympathy, Disgust, Contempt	Embarrassment	Fear, Sympathy, Disgust, Contempt
26	NV SUP	0.53	0	0.15	0.42	0.15	0.35	0.26	0	0	0	0.57	0.6	0	0.6	0	0	0.96	0	Relief, Joy, amusement, Awe, Contempt	Relief, Joy, amusement	Awe, Contempt
27	NV SUP	0.28	0	0.21	0.81	0.21	0.28	0.28	0.11	0.11	0	0.21	0.67	0	0.85	0.11	0	0.88	0.11	Relief, Contempt, Content, Amusement, Boredom	Relief	Contempt, Content, Amusement, Boredom
28	NV SYM	0	0	0	0	0.52	0.52	0.34	0.2	0.48	0.2	0	0.7	0.2	0.44	0.78	0.4	0	0.91	Sadness, Boredom, Contempt, Embarrassment, Relief	Sadness	Boredom, Contempt, Embarrassment, Relief
29	NV SYM	0	0	0	0	0.7	0.36	0.22	0.3	0.12	0	0.12	0.65	0	0.12	0.87	0.46	0	0.91	Sadness, Boredom, Shame, Contempt, Disgust	Sadness	Boredom, Shame, Contempt, Disgust
30	NV SYM	0.11	0	0.11	0.2	0.39	0.34	0	0	0	0.11	0.11	0.2	0	0.2	0.94	0.52	0	0.89	Shame, Boredom, contempt, relief, Awe	Shame	Boredom, contempt, relief, Awe
31	NV SYM	0	0.24	0.77	0.24	0.13	0	0.24	0	0.13	0.45	0	0.39	0	0.32	0.86	0.24	0.24	0.9	Sadness, Anxiety, Fear, Relief, Shame	Sadness, Anxiety	Fear, Relief, Shame
32	V AMU	0.83	0.12	0.22	0.22	0.36	0	0.66	0.12	0.12	0.12	0.88	0.82	0.29	0.12	0.12	0.22	0.12	0	Content, Relief, Anxiety, Embarrassment, Fear	Content	Relief, Anxiety, Embarrassment, Fear
33	V AMU	0.82	0	0	0.34	0.11	0	0.57	0	0	0	0.96	0.34	0.11	0.11	0	0	0.2	0.11	Content, Relief, Anxiety, Embarrassment, Fear	Content	Relief, Anxiety, Embarrassment, Fear
34	V ANG	0	0.9	0.34	0.28	0.2	0.79	0.28	0.51	0.2	0	0.2	0.71	0.51	0.44	0.11	0	0.2	0.44	Disgust, Pride, Relief, Sympathy, Sadness	Disgust	Pride, Relief, Sympathy, Sadness
35	V ANG	0.2	0.84	0.43	0.11	0.43	0.83	0.43	0.71	0.2	0.39	0.11	0.59	0.27	0.33	0.27	0	0.11	0.47	Disgust, Anxiety, Boredom, Fear, Joy	Disgust	Anxiety, Boredom, Fear, Joy
36	V BOR	0.12	0.12	0	0.21	0.96	0.21	0	0.28	0.21	0.21	0.12	0.75	0.21	0.12	0.67	0.28	0	0.12	Sadness, Contempt, Relief, Anger, Amusement	Sadness	Contempt, Relief, Anger, Amusement
37	V BOR	0.1	0.31	0	0	0.95	0.45	0.1	0.36	0.18	0.1	0.25	0.57	0	0.1	0.81	0.25	0	0.1	Sadness, Shame, Joy, Embarrassment, Contempt	Sadness	Shame, Joy, Embarrassment, Contempt
38	V CON	0.64	0	0.11	0.48	0.11	0.28	0.84	0	0.2	0.11	0.82	0.87	0.2	0.2	0.2	0.11	0.11	0.11	Amusement, Awe, Contempt, Pride, Relief	Amusement, Awe	Contempt, Pride, Relief
39	V CON	0.21	0.12	0.21	0.29	0.36	0.46	0.81	0.12	0	0	0.62	0.93	0.41	0.29	0.29	0.12	0.12	0	Joy, Boredom, Contempt, Pride, Sadness	Joy	Boredom, Contempt, Pride, Sadness
40	V DIS	0.12	0.53	0.28	0	0.87	0.81	0.21	0.73	0.12	0.12	0.4	0.69	0.21	0.28	0.45	0.12	0	0.12	Anger, Anxiety, Pride, Relief, Sadness	Anger	Anxiety, Pride, Relief, Sadness
41	V DIS	0.2	0.44	0.11	0.2	0.62	0.75	0.6	0.57	0	0	0.11	0.93	0.11	0	0.2	0.11	0	0.44	Boredom, Sadness, Sympathy, Awe, Amusement	Boredom	Sadness, Sympathy, Awe, Amusement
42	V EMB	0.1	0	0.86	0.36	0.31	0	0.44	0	0.66	0.81	0.1	0.73	0.25	0.25	0.1	0.18	0.69	0.18	Surprise, Content, Boredom, Awe, Shame	Surprise	Content, Boredom, Awe, Shame
43	V EMB	0.89	0.11	0.67	0.34	0.11	0.11	0.34	0.11	0.64	0.39	0.77	0.27	0.11	0.48	0.34	0.11	0.51	0.11	Relief, Surprise, Fear, Content, Awe	Relief, Surprise	Fear, Content, Awe
44	V FEA	0.37	0	0.78	0	0.1	0	0.19	0.19	0.37	0.9	0.19	0.46	0.1	0.32	0.81	0.19	0.49	0.19	Anxiety, Sadness, Surprise, Embarrassment, Relief	Anxiety, Sadness	Surprise, Embarrassment, Relief

Item #	Type	Amu	Ang	Anx	Awe	Bor	Cmp	Con	Dis	Emb	Fea	Joy	Neu	Pri	Rel	Sad	Sha	Sup	Sym	Selected Distractors	Partially correct distractor	Incorrect distractor
45	V FEA	0.1	0.1	0.77	0.32	0.37	0.26	0.49	0.1	0.52	0.73	0.37	0.84	0.1	0.37	0.37	0.32	0.41	0.57	Sympathy, Embarrassment, Sadness, Content, Boredom	Sympathy, Embarrassment	Sadness, Content, Boredom
46	V SAD	0	0	0.6	0	0.28	0.11	0.11	0.2	0.34	0.71	0	0.68	0	0.34	0.93	0.34	0.2	0.64	Fear, Anxiety, Sympathy, Disgust, Boredom	Fear, Anxiety, Sympathy	Disgust, Boredom
47	V SAD	0	0	0.35	0.11	0.87	0.28	0.28	0	0.35	0.28	0	0.81	0	0.11	0.87	0.52	0.11	0.28	Boredom, Shame, Sympathy, Anxiety, Content	Boredom	Shame, Sympathy, Anxiety, Content
48	V SUP	0.65	0	0.19	0.46	0	0.19	0.5	0	0	0	0.85	0.38	0.38	0.56	0	0	0.91	0.19	Joy, Relief, Anxiety, Pride, Anger	Joy, Relief	Anxiety, Pride, Anger
49	V SUP	0.81	0.19	0.11	0.53	0	0.38	0.33	0.33	0	0.11	0.81	0.46	0.19	0.33	0	0	0.89	0.11	Joy, Awe, Contempt, Content, Fear	Joy, Awe	Contempt, Content, Fear
50	V SYM	0.21	0.11	0.49	0.21	0.49	0.4	0.4	0.21	0.28	0.35	0.11	0.78	0	0.28	0.52	0.11	0.21	0.93	Sadness, Anxiety, Boredom, Contempt, Content	Sadness, Anxiety, Boredom	Contempt, Content
51	V SYM	0.11	0.11	0.34	0.4	0	0.34	0.52	0.11	0.28	0.4	0.55	0.79	0.28	0.64	0.2	0.2	0.68	0.85	Surprise, Embarrassment, Disgust, Pride, Sadness	Surprise	Embarrassment, Disgust, Pride, Sadness
52	C ANGxCENT																					
	Concealed	0.89	0.48	0	0.18	0.1	0.45	0.69	0.36	0.1	0.1	0.85	0.54	0.31	0.25	0.18	0	0.1	0.31	Content, Disgust, Embarrassment, Sadness	Wildcard + Content; Anger + Wildcard; Content + Anger	All other answers
	Displayed	0.45	0.93	0.18	0.00	0.36	0.69	0.18	0.57	0.18	0.31	0.61	0.25	0.18	0.36	0.54	0.10	0.10	0.00	Anger, Disgust, Embarrassment, Sadness		
53	C ANGxCENT																					
	Concealed	0.25	0.25	0.1	0	0.69	0.36	0.76	0.25	0.1	0.1	0.48	0.81	0.1	0.78	0.51	0.31	0.18	0.63	Content, Anxiety, Joy, Sympathy	Wildcard + Content; Anger + Wildcard; Content + Anger	All other answers
	Displayed	0.00	0.54	0.56	0.10	0.80	0.36	0.18	0.66	0.40	0.36	0.25	0.36	0.00	0.40	0.86	0.40	0.36	0.36	Anger, Anxiety, Joy, Sympathy		
54	C ANGxCENT																					
	Concealed	0.66	0.38	0.1	0.26	0.32	0.38	0.83	0.1	0.19	0	0.89	0.73	0.19	0.42	0.19	0	0	0.1	Content, Amusement, Anxiety, Sadness		
	Displayed	0.64	0.91	0.58	0.10	0.32	0.38	0.00	0.32	0.19	0.32	0.56	0.42	0.00	0.32	0.68	0.42	0.26	0.10	Anger, Amusement, Anxiety, Sadness		
55	C ANGxCENT																					
	Concealed	0.43	0.29	0.24	0.09	0.59	0.39	0.87	0.24	0.09	0.09	0.76	0.73	0.39	0.72	0.09	0.17	0	0.09	Content, Anxiety, Relief, Shame	Wildcard + Content; Anger + Wildcard; Content + Anger	All other answers
	Displayed	0.17	0.79	0.49	0.09	0.75	0.80	0.24	0.82	0.29	0.09	0.29	0.29	0.00	0.24	0.29	0.46	0.09	0.09	Anger, Anxiety, Relief, Shame		

Item #	Type	Amu	Ang	Anx	Awe	Bor	Cmp	Con	Dis	Emb	Fea	Joy	Neu	Pri	Rel	Sad	Sha	Sup	Sym	Selected Distractors	Partially correct distractor	Incorrect distractor
56	C ANGxCENT																					
	Concealed	0.46	0.54	0.23	0	0.17	0.42	0.75	0.23	0	0	0.64	0.94	0.29	0.52	0.17	0	0	0	Content, Relief, Sadness, Disgust	Wildcard + Content; Anger + Wildcard; Content + Anger	All other answers
	Displayed	0.54	0.86	0.00	0.00	0.54	0.78	0.42	0.75	0.09	0.23	0.60	0.42	0.29	0.09	0.49	0.09	0.09	0.00	Anger, Relief, Sadness, Disgust		
57	C CENTxANG																					
	Concealed	0.69	0.76	0.09	0.16	0.09	0.6	0.58	0.55	0.37	0.09	0.84	0.69	0.28	0.16	0.23	0.23	0.16	0.23	Anger, Disgust, Embarrassment, Sadness	Wildcard + Content; Anger + Wildcard; Content + Anger	All other answers
	Displayed	0.70	0.92	0.09	0.28	0.00	0.77	0.16	0.69	0.23	0.16	0.65	0.16	0.09	0.09	0.00	0.09	0.00	0.00	Content, Disgust, Embarrassment, Sadness		
58	C JOYxSAD																					
	Concealed	0.81	0.1	0	0.1	0.26	0.37	0.45	0.45	0.32	0.19	0.85	0.57	0.19	0.41	0.67	0.41	0.26	0.52	Sadness, Sympathy, Shame, Awe	Joy + Wildcard; Wildcard +Sadness; Sadness + Joy	All other answers
	Displayed	0.70	0.10	0.26	0.00	0.10	0.26	0.10	0.55	0.52	0.26	0.55	0.32	0.10	0.41	0.93	0.57	0.10	0.10	Joy, Sympathy, Shame, Awe		
59	C JOYxSAD																					
	Concealed	0.23	0.37	0.16	0	0.41	0.33	0.67	0.28	0.33	0.16	0.64	0.88	0.09	0.37	0.79	0.16	0.28	0.44	Sadness, Anxiety, Fear, Shame	Joy + Wildcard; Wildcard +Sadness; Sadness + Joy	All other answers
	Displayed	0.78	0.41	0.48	0.16	0.33	0.28	0.28	0.33	0.00	0.55	0.84	0.33	0.23	0.28	0.78	0.50	0.23	0.16	Joy, Anxiety, Fear, Shame		
60	C SADxJOY																					
	Concealed	0.68	0.24	0	0	0	0.17	0.72	0.17	0.24	0	0.95	0.39	0.24	0.43	0.09	0	0	0.55	Joy, Disgust, Contempt, Awe	Sadness + Wildcard; Wildcard + Joy; Joy + Sadness	All other answers
	Displayed	0.24	0.35	0.47	0.00	0.47	0.43	0.24	0.73	0.30	0.47	0.39	0.00	0.00	0.24	0.94	0.00	0.00	0.50	Sadness, Disgust, Contempt, Awe		
61	C SADxJOY																					
	Concealed	0.79	0	0	0.1	0.1	0	0.64	0.1	0.1	0	0.96	0.25	0	0.36	0.1	0.1	0.31	0	Joy, Embarrassment, Relief, Surprise	Sadness + Wildcard; Wildcar	All other answers

Item #	Type	Amu	Ang	Anx	Awe	Bor	Cmp	Con	Dis	Emb	Fea	Joy	Neu	Pri	Rel	Sad	Sha	Sup	Sym	Selected Distractors	Partially correct distractor	Incorrect distractor
																					d + Joy; Joy + Sadness	
	Displayed	0.36	0.10	0.69	0.10	0.51	0.25	0.40	0.10	0.61	0.44	0.48	0.36	0.00	0.25	0.92	0.44	0.31	0.00	Sadness, Embarrassment, Relief, Surprise		
62	C SADxJOY																					
	Concealed	0.8	0.11	0	0.27	0.33	0.27	0.68	0.27	0.2	0.2	0.91	0.63	0.2	0.33	0.33	0.11	0.38	0.11	Joy, Anxiety, Fear, Relief	Sadness + Wildcard; Wildcard + Joy; Joy + Sadness	All other answers
	Displayed	0.33	0.20	0.67	0.00	0.83	0.38	0.33	0.27	0.43	0.53	0.61	0.38	0.00	0.27	0.78	0.50	0.47	0.20	Sadness, Anxiety, Fear, Relief		
63	C SADxJOY																					
	Concealed	0.43	0.11	0.27	0.11	0.27	0.39	0.64	0.27	0.27	0.33	0.94	0.27	0	0.51	0.7	0.11	0.11	0.2	Joy, Relief, Anxiety, Fear	Sadness + Wildcard; Wildcard + Joy; Joy + Sadness	All other answers
	Displayed	0.43	0.11	0.69	0.00	0.00	0.27	0.20	0.43	0.43	0.64	0.74	0.11	0.11	0.20	0.93	0.27	0.00	0.00	Sadness, Relief, Anxiety, Fear		
64	C SADxJOY																					
	Concealed	0.65	0.1	0	0	0	0.26	0.81	0	0.32	0.1	0.91	0.72	0	0.45	0.45	0.32	0	0.1	Joy, anxiety, Fear, Pride	Sadness + Wildcard; Wildcard + Joy; Joy + Sadness	All other answers
	Displayed	0.00	0.19	0.67	0.00	0.32	0.19	0.32	0.26	0.68	0.57	0.10	0.37	0.19	0.26	0.89	0.80	0.19	0.19	Sadness, Anxiety, fear, pride		

Note:

Table S2 Study 2 Item-level Scoring and Confusions

Item #	Type	Selected Distractors	Correct answer (*pi*)	Partially correct distractors (*pi*)	Incorrect distractors (*pi*)
1	NV AMU	Joy, Content, Relief, Pride, Surprise	Amusement (.91)	Joy (.59)	Content (.17) Relief (.08) Pride (.15) Surprise (.07)
2	NV AMU	Joy, Pride, Content, Contempt, Surprise	Amusement (.89)	Joy (.40)	Pride (.32) Content (.31) Contempt (.18) Surprise (.21)
3	NV AMU	Joy, Content, Relief, Contempt, Awe	Amusement (.89)	Joy (.42)	Content (.31) Relief (.25) Contempt (.23) Awe (.20)
4	NV AMU	Joy, Relief, Content, Surprise, Embarrassment	Amusement (0.78)	Joy (0.78)	Joy (0.78) Relief (0.25) Content (0.21) Surprise (0.19) Embarrassment (0.07)
5	NV AMU	Joy, Content, Relief, Pride, Embarrassment	Amusement (0.85)	Joy (0.7)	Content (0.19) Relief (0.08) Pride (0.27) Embarrassment (0.06)
6	NV AMU	Joy, Relief, Content, Surprise, Fear	Amusement (0.85)	Joy (0.72)	Relief (0.12) Content (0.22) Surprise (0.14) Fear (0.04)
7	NV BOR	Sadness, Disgust, Shame, Anxiety, Contempt	Boredom (0.91)	Sadness (0.35) Disgust (0.22)	Shame (0.25) Anxiety (0.15) Contempt (0.33)
8	NV BOR	Contempt, Embarrassment, Disgust, Anxiety, Content	Boredom (0.9)	Contempt (0.33) Embarrassment (0.35)	Disgust (0.21) Anxiety (0.25) Content (0.25)
9	NV BOR	Anxiety, Shame, Contempt, Disgust, relief	Boredom (0.82)	Anxiety (0.5) Shame (0.48)	Contempt (0.25) Disgust (0.28) Relief (0.24)
10	NV CON	Joy, Amusement, Contempt, Pride, Boredom	Content (0.84)	Joy (0.4) Amusement (0.39)	Contempt (0.22) Pride (0.46) Boredom (0.23)
11	NV CON	Relief, Joy, Amusement, Contempt, Awe	Content (0.81)	Relief (0.59) Joy (0.42) Amusement (0.32)	Contempt (0.23) Awe (0.17)
12	NV CON	Joy, Contempt, Anxiety, Sadness, Amusement	Content (0.88)	Joy (0.3)	Contempt (0.31) Anxiety (0.33) Sadness (0.29) Amusement (0.29)
13	NV CON	Joy, Amusement, Relief, Awe, Contempt	Content (0.85)	Joy (0.48) Amusement (0.32) Relief (0.35)	Awe (0.22) Contempt (0.29)

Item #	Type	Selected Distractors	Correct answer (*pi)*	Partially correct distractors (*pi*)	Incorrect distractors (*pi*)
14	NV DIS	Contempt, Anger, Sadness, Shame, Fear	Disgust (0.93)	Contempt (0.39)	Anger (0.29) Sadness (0.19) Shame (0.13) Fear (0.05)
15	NV DIS	Sadness, Shame, Fear, Contempt, Anger	Disgust (0.86)	Sadness (0.58)	Shame (0.23) Fear (0.29) Contempt (0.21) Anger (0.19)
16	NV EMB	Amusement, Sadness, Anxiety, Sympathy, Fear	Embarrassmen t (0.88)	Amusement (0.32) Sadness (0.37)	Anxiety (0.35) Sympathy (0.29) Fear (0.14)
17	NV EMB	Amusement, Shame, Fear, Surprise, Joy	Embarrassmen t (0.84)	Amusement (0.48) Shame (0.33) Fear (0.37)	Surprise (0.35) Joy (0.18)
18	NV EMB	Shame, Amusement, Sadness, Anxiety, Disgust	Embarrassmen t (0.86)	Shame (0.42) Amusement (0.52)	Sadness (0.14) Anxiety (0.25) Disgust (0.23)
19	NV REL	Boredom, Sympathy, Awe, Anxiety, Contempt	Relief (0.94)	Boredom (0.34)	Sympathy (0.1) Awe (0.2) Anxiety (0.2) Contempt (0.12)
20	NV REL	Anxiety, Boredom, Sadness, Disgust, Fear	Relief (0.96)	Anxiety (0.24)	Boredom (0.22) Sadness (0.14) Disgust (0.1) Fear (0.08)
21	NV SAD	Shame, Sympathy, Fear, Anxiety, Embarrassment	Sadness (0.91)	Shame (0.33)	Sympathy (0.36) Fear (0.12) Anxiety (0.22) Embarrassment (0.24)
22	NV SAD	Sympathy, Disgust, Fear, Anxiety, Shame	Sadness (0.85)	Sympathy (0.43)	Disgust (0.24) Fear (0.25) Anxiety (0.42) Shame (0.29)
23	NV SHA	Embarrassment, Sadness, Contempt, Disgust, Fear	Shame (0.85)	Embarrassment (0.67)	Sadness (0.38) Contempt (0.15) Disgust (0.13) Fear (0.07)
24	NV SHA	Embarrassment, Boredom, Sympathy, Contempt, Anxiety	Shame (0.81)	Embarrassment (0.47)	Boredom (0.37) Sympathy (0.41) Contempt (0.29) Anxiety (0.32)
25	NV SHA	Embarrassment, Fear, Sympathy, Disgust, Contempt	Shame (0.87)	Embarrassment (0.56)	Fear (0.17) Sympathy (0.42) Disgust (0.14) Contempt (0.14)
26	NV SUP	Relief, Joy, amusement, Awe, Contempt	Surprise (0.89)	Relief (0.38) Joy (0.24) Amusement (0.3)	Awe (0.34) Contempt (0.19)
27	NV SUP	Relief, Contempt, Content, Amusement, Boredom	Surprise (0.88)	Relief (0.6)	Contempt (0.14) Content (0.26) Amusement (0.13) Boredom (0.15)

Item #	Type	Selected Distractors	Correct answer (*pi)*	Partially correct distractors (*pi*)	Incorrect distractors (*pi*)
28	NV SYM	Sadness, Boredom, Contempt, Embarrassment, Relief	Sympathy (0.87)	Sadness (0.47)	Boredom (0.22) Contempt (0.31) Embarrassment (0.27) Relief (0.25)
29	NV SYM	Sadness, Boredom, Shame, Contempt, Disgust	Sympathy (0.82)	Sadness (0.6)	Boredom (0.39) Shame (0.32) Contempt (0.2) Disgust (0.14)
30	NV SYM	Shame, Boredom, contempt, relief, Awe	Sympathy (0.89)	Shame (0.4)	Boredom (0.39) Contempt (0.23) Relief (0.19) Awe (0.2)
31	NV SYM	Sadness, Anxiety, Fear, Relief, Shame	Sympathy (0.77)	Sadness (0.6) Anxiety (0.55)	Fear (0.29) Relief (0.19) Shame (0.2)
32	V AMU	Content, Relief, Anxiety, Embarrassment, Fear	Amusement (0.75)	Content (0.72)	Relief (0.36) Anxiety (0.32) Embarrassment (0.24) Fear (0.12)
33	V AMU	Content, Relief, Anxiety, Embarrassment, Fear	Amusement (0.88)	Content (0.63)	Relief (0.29) Anxiety (0.13) Embarrassment (0.12) Fear (0.06)
34	V ANG	Disgust, Pride, Relief, Sympathy, Sadness	Anger (0.85)	Disgust (0.46)	Pride (0.46) Relief (0.25) Sympathy (0.3) Sadness (0.07)
35	V ANG	Disgust, Anxiety, Boredom, Fear, Joy	Anger (0.67)	Disgust (0.67)	Anxiety (0.48) Boredom (0.41) Fear (0.2) Joy (0.29)
36	V BOR	Sadness, Contempt, Relief, Anger, Amusement	Boredom (0.87)	Sadness (0.51)	Contempt (0.45) Relief (0.21) Anger (0.15) Amusement (0.1)
37	V BOR	Sadness, Shame, Joy, Embarrassment, Contempt	Boredom (0.82)	Sadness (0.65)	Shame (0.32) Joy (0.15) Embarrassment (0.16) Contempt (0.31)
38	V CON	Amusement, Awe, Contempt, Pride, Relief	Content (0.82)	Amusement (0.38) Awe (0.39)	Contempt (0.35) Pride (0.38) Relief (0.31)
39	V CON	Joy, Boredom, Contempt, Pride, Sadness	Content (0.84)	Joy (0.26)	Boredom (0.31) Contempt (0.46) Pride (0.48) Sadness (0.11)
40	V DIS	Anger, Anxiety, Pride, Relief, Sadness	Disgust (0.76)	Anger (0.5)	Anxiety (0.28) Pride (0.33) Relief (0.43) Sadness (0.44)
41	V DIS	Boredom, Sadness, Sympathy, Awe, Amusement	Disgust (0.68)	Boredom (0.65)	Sadness (0.33) Sympathy (0.47) Awe (0.4) Amusement (0.23)
42	V EMB	Surprise, Content, Boredom, Awe, Shame	Embarrassmen t (0.64)	Surprise (0.7)	Content (0.44) Boredom (0.15) Awe (0.36) Shame (0.44)

Item #	Type	Selected Distractors	Correct answer (*pi)*	Partially correct distractors (*pi*)	Incorrect distractors (*pi*)
43	V EMB	Relief, Surprise, Fear, Content, Awe	Embarrassment (0.67)	Relief (0.27) Surprise (0.72)	Fear (0.2) Content (0.36) Awe (0.45)
44	V FEA	Anxiety, Sadness, Surprise, Embarrassment, Relief	Fear (0.72)	Anxiety (0.58) Sadness (0.47)	Surprise (0.45) Embarrassment (0.25) Relief (0.31)
45	V FEA	Sympathy, Embarrassment, Sadness, Content, Boredom	Fear (0.67)	Sympathy (0.45) Embarrassment (0.47)	Sadness (0.27) Content (0.66) Boredom (0.25)
46	V SAD	Fear, Anxiety, Sympathy, Disgust, Boredom	Sadness (0.82)	Fear (0.44) Anxiety (0.34) Sympathy (0.53)	Disgust (0.13) Boredom (0.3)
47	V SAD	Boredom, Shame, Sympathy, Anxiety, Content	Sadness (0.72)	Boredom (0.64)	Shame (0.44) Sympathy (0.28) Anxiety (0.25) Content (0.37)
48	V SUP	Joy, Relief, Anxiety, Pride, Anger	Surprise (0.87)	Joy (0.59) Relief (0.26)	Anxiety (0.26) Pride (0.25) Anger (0.05)
49	V SUP	Joy, Awe, Contempt, Content, Fear	Surprise (0.83)	Joy (0.62) Awe (0.37)	Contempt (0.17) Content (0.26) Fear (0.19)
50	V SYM	Sadness, Anxiety, Boredom, Contempt, Content	Sympathy (0.82)	Sadness (0.51) Anxiety (0.27) Boredom (0.3)	Contempt (0.32) Content (0.37)
51	V SYM	Surprise, Embarrassment, Disgust, Pride, Sadness	Sympathy (0.82)	Surprise (0.55)	Embarrassment (0.31) Disgust (0.13) Pride (0.3) Sadness (0.44)
52	C ANGxCENT		Concealed anger displayed content (.91)	Wildcard + Content or Anger + Wildcard (.48); Content + Anger (.42)	All other answers
	Concealed	Content, Disgust, Embarrassment, Sadness			
	Displayed	Anger, Disgust, Embarrassment, Sadness			
53	C ANGxCENT		Concealed anger	Wildcard + Content or Anger + Wildcard (.48); Content + Anger (.18)	All other answers

Item #	Type	Selected Distractors	Correct answer (*pi)*	Partially correct distractors (*pi*)	Incorrect distractors (*pi*)
			displayed content (.72)		
	Concealed	Content, Anxiety, Joy, Sympathy			
	Displayed	Anger, Anxiety, Joy, Sympathy			
54	C ANGxCENT		Concealed anger displayed content (.84)	Wildcard + Content or Anger + Wildcard (.48); Content + Anger (.15)	All other answers
	Concealed	Content, Amusement, Anxiety, Sadness			
	Displayed	Anger, Amusement, Anxiety, Sadness			
55	C ANGxCENT		Concealed anger displayed content (.83)	Wildcard + Content or Anger + Wildcard (.47); Content + Anger (.15)	All other answers
	Concealed	Content, Anxiety, Relief, Shame			
	Displayed	Anger, Anxiety, Relief, Shame			
56	C ANGxCENT		Concealed anger displayed content (.86)	Wildcard + Content or Anger + Wildcard (.48); Content + Anger (.26)	All other answers
	Concealed	Content, Relief, Sadness, Disgust			
	Displayed	Anger, Relief, Sadness, Disgust			
57	C CENTxANG		Concealed content displayed anger (.54)	Content + wildcard or Wildcard+ Anger (.44); Anger + Content (.87)	All other answers
	Concealed	Anger, Disgust, Embarrassment, Sadness			

Item #	Type	Selected Distractors	Correct answer (*pi*)	Partially correct distractors (*pi*)	Incorrect distractors (*pi*)
	Displayed	Content, Disgust, Embarrassment, Sadness			
58	C JOYxSAD		Concealed joy displayed sadness (.58)	Joy + Wildcard or Wildcard +Sadness (.44); Sadness + Joy (.87)	All other answers
	Concealed	Sadness, Sympathy, Shame, Awe			
	Displayed	Joy, Sympathy, Shame, Awe			
59	C JOYxSAD		Concealed joy displayed sadness (.78)	Joy + Wildcard or Wildcard +Sadness; Sadness (.46)+ Joy (.73)	All other answers
	Concealed	Sadness, Anxiety, Fear, Shame			
	Displayed	Joy, Anxiety, Fear, Shame			
60	C SADxJOY		Concealed sadness displayed joy (.93)	Sadness + Wildcard or Wildcard + Joy (.47); Joy + Sadness (.25)	All other answers
	Concealed	Joy, Disgust, Contempt, Awe			
	Displayed	Sadness, Disgust, Contempt, Awe			
61	C SADxJOY		Concealed sadness displayed joy (.89)	Sadness + Wildcard or Wildcard + Joy (.48); Joy + Sadness (.15)	All other answers
	Concealed	Joy, Embarrassment, Relief, Surprise			
	Displayed	Sadness, Embarrassment, Relief, Surprise			
62	C SADxJOY		Concealed sadness displayed joy (.85)	Sadness + Wildcard or Wildcard + Joy (.47); Joy + Sadness (.29)	All other answers

Item #	Type	Selected Distractors	Correct answer (*pi*)	Partially correct distractors (*pi*)	Incorrect distractors (*pi*)
	Concealed	Joy, Anxiety, Fear, Relief			
	Displayed	Sadness, Anxiety, Fear, Relief			
63	C SADxJOY		Concealed sadness displayed joy (.92)	Sadness + Wildcard or Wildcard + Joy (.46); Joy + Sadness (.64)	All other answers
	Concealed	Joy, Relief, Anxiety, Fear			
	Displayed	Sadness, Relief, Anxiety, Fear			
64	C SADxJOY		Concealed sadness displayed joy (.92)	Sadness + Wildcard or Wildcard + Joy (.47); Joy + Sadness (.29)	All other answers
	Concealed	Joy, anxiety, Fear, Pride			
	Displayed	Sadness, Anxiety, fear, pride			

Note:

Appendix

Emotion Themes

Instructions: The table below contains 18 emotion words. Each emotion word has a theme which describes a 'script' associated with experience of that emotion. **Please read each emotion theme below:**

Emotion	Themes
Amusement	Playful with others in the environment
Anger	A demeaning offense against me and mine
Anxiety	Facing an uncertain existential threat
Awe	Feeling small relative to the environment/others; situation challenged worldview
Boredom	Low effort, low attention, and desire to shut out what is happening
Contempt	Evaluating that others are unworthy; experiencing revulsion toward others
Content	Appreciation of present circumstance; feeling satisfied and secure
Disgust	Taking in or being too close to an indigestible object or idea (metaphorically speaking)
Embarrassment	Being the target of an unexpected or accidental experience that is amusing to others
Fear	Facing an immediate and concrete physical danger
Joy	Improvement in resources; increased positive energy
Neutral	The lack of feeling any emotion
Pride	Accomplishment; able to take on new challenges
Relief	A distressing goal-incongruent condition that has changed for the better
Sadness	Having experienced an irrevocable loss
Shame	Having failed to live up to an ego-ideal
Surprise	Experiencing something unexpected; feeling stunned
Sympathy	Being moved by another's suffering and wanting to help

Autobiographical recall prompt Study 1

"Begin by imagining the situation as vividly as you can; picture the events happening to you and see all the details of the situation. Please explain what events preceded the emotion and why you felt the emotion. Please only write examples of events that actually happened to you; we will later ask you to reenact the emotions from these events during the film shoot. We encourage you to write stories that are personally meaningful so this packet will not be collected (i.e. only you will see the stories you write here)."

Method Acting Prompt Study 1

"I want you to go back to the time you felt a slight/medium amount of [emotion]. Imagining the situation as vividly as you can; picture the events happening to you and see all the details of the situation. When you are ready, please show me [emotion]."

Materials- MET Instructions

Nonverbal and Verbal items

"On the following pages you will see short video clips of people expressing a variety of emotions. Please watch each video clip carefully (they are very short) and then answer the questions."

The text for the response prompt was as follows:

"What emotion did the person express?"

Concealed emotion items

The concealed emotion stimuli were presented in separate trials with unique instructions. Furthermore, the concealed emotions included different instructions that explained the unique nature of the task to participants. The instructions were as follows:

"On the following pages you will see 13 short video clips of people expressing a variety of emotions. Each person will try to hide one emotion by expressing a different emotion. Please watch each video clip carefully (they are very short) and then fill in the blanks in the following sentence:

"I think the person tried to hide ________ by expressing ________."

For each concealed emotion item, participants were allowed to select one label for the emotion they believed was expressed, and one label for the emotion they believe was concealed.
